# Progesterone attenuates Th17-cell pathogenicity in autoimmune uveitis via Id2/Pim1 axis

**DOI:** 10.1186/s12974-023-02829-3

**Published:** 2023-06-21

**Authors:** Xiuxing Liu, Chenyang Gu, Jianjie Lv, Qi Jiang, Wen Ding, Zhaohao Huang, Yidan Liu, Yuhan Su, Chun Zhang, Zhuping Xu, Xianggui Wang, Wenru Su

**Affiliations:** 1 State Key Laboratory of Ophthalmology, Zhongshan Ophthalmic Center, Sun Yat-Sen University, Guangdong Provincial Key Laboratory of Ophthalmology and Visual Science, Guangzhou, 510060 China; 2 Guangzhou Women and Children’s Medical Center, Guangzhou Medical University, Guangzhou, 510623 China; 3 Department of Clinical Medicine, Zhongshan School of Medicine, Sun Yat-Sen University, Guangzhou, 510060 China; 4 Department of Ophthalmology, West China Hospital, Sichuan University, Chengdu, 610041 Sichuan China; 5 Eye Center of Xiangya Hospital, Central South University, Changsha, 410078 China; 6 Hunan Key Laboratory of Ophthalmology, Xiangya Hospital, Central South University, Changsha, 410078 China

**Keywords:** Progesterone, Autoimmune uveitis, Single-cell RNA sequencing, Th17 cells, Id2, Treg

## Abstract

**Background:**

Autoimmune uveitis (AU) is the most common ophthalmic autoimmune disease (AD) and is characterized by a complex etiology, high morbidity, and high rate of blindness. AU remission has been observed in pregnant female patients. However, the effects of progesterone (PRG), a critical hormone for reproduction, on the treatment of AU and the regulatory mechanisms remain unclear.

**Methods:**

To this end, we established experimental autoimmune uveitis (EAU) animal models and constructed a high-dimensional immune atlas of EAU-model mice undergoing PRG treatment to explore the underlying therapeutic mechanisms of PRG using single-cell RNA sequencing.

**Results:**

We found that PRG ameliorated retinal lesions and inflammatory infiltration in EAU-model mice. Further single-cell analysis indicated that PRG reversed the EAU-induced expression of inflammatory genes (AP-1 family, S100a family, and *Cxcr4*) and pathological processes related to inflammatory cell migration, activation, and differentiation. Notably, PRG was found to regulate the Th17/Treg imbalance by increasing the reduced regulatory functional mediators of Tregs and diminishing the overactivation of pathological Th17 cells. Moreover, the Id2/Pim1 axis, IL-23/Th17/GM-CSF signaling, and enhanced Th17 pathogenicity during EAU were reversed by PRG treatment, resulting in the alleviation of EAU inflammation and treatment of AD.

**Conclusions:**

Our study provides a comprehensive single-cell map of the immunomodulatory effects of PRG therapy on EAU and elaborates on the possible therapeutic mechanisms, providing novel insights into its application for treating autoimmune diseases.

**Supplementary Information:**

The online version contains supplementary material available at 10.1186/s12974-023-02829-3.

## Background

Autoimmune diseases (ADs) are defined as a set of chronic diseases of immune homeostasis disruption, characterized by a complex etiology, high morbidity, and mortality. Their refractory nature induces obvious burdens on medical care utilization and high socioeconomic costs. As the most common ocular AD, autoimmune uveitis (AU) represents a group of sight-threatening intraocular inflammatory diseases characterized by the disruption of intraocular immune homeostasis and sustained inflammation [1, 2]. Experimental autoimmune uveitis (EAU) is a prototypic organ-specific animal model of human endogenous uveitis induced by immunization with retinal antigens [3]. Emerging research has boosted our understanding of the pro-inflammatory cellular and molecular basis that promotes AU and EAU, incorporating pathogenic CD4 + T helper 17 cell (Th17)/Th1 infiltration [4] and Th17/regulatory T cell (Treg) disequilibrium [5]. Nevertheless, the explicit mechanisms underlying immune interactions in AU remain elusive, and novel therapy with both superior efficacy and fewer adverse effects are required to benefit patients with refractory AU.

There is evidence of sexual dimorphism in the immune system. For example, there are prominent sex differences in the incidence of AD. Generally, females have a greater prevalence of multiple AD than men, incorporating multiple sclerosis (MS) [6] and systemic lupus erythematosus [7]. Therefore, hormonal factors presumably participate in modulating the course of AD, and sex hormones, such as estrogens, progesterone, prolactin, and androgens, probably play a role in this. Indeed, under physiological conditions, such as puberty, pregnancy, and menopause, hormones affect the course and frequency of the disease. For example, it has been clinically observed that female patients with AD experience remission during pregnancy and relapse after childbirth [8]. More importantly, alterations in progesterone (PRG) levels during gestation were found to coincide with the temporary improvement and worsening of several ADs, such as MS [9] and rheumatoid arthritis (RA) [10]. Gestation introduces a unique immune condition, as the maternal immune system is capable of tolerating the presence of a semi-allogenic fetus [11, 12], which mostly occurs due to the levels of PRG during pregnancy. In conclusion, hormone levels exhibit a strong correlation with the effects of AD, which could be expected to play a role in its pathogenesis and treatment.

PRG is crucial for the establishment and maintenance of normal functions at multiple stages of mammalian reproduction, both through its endocrine and immunological effects [13]. Beyond the maintenance of pregnancy, PRG has been reported to exert neuroprotective effects in promoting myelin formation and the maturation of developing fetal brain [14, 15]. As such, several clinical studies have reported the safety and efficacy of PRG for traumatic brain injury and motoneuron degeneration [16–18]. Notably, recent studies found that PRG also has immunomodulatory functions, suggesting its therapeutic role in autoimmune inflammation. High levels of PRG dampen CD4 + T cell activation and several disease-relevant markers, such as STAT1/3 and their downstream targets [19]. Further, PRG switches the immune reaction from pro-inflammatory to anti-inflammatory, facilitating Treg cell differentiation and the downregulation of IFN-γ generated by natural killer (NK) cells [20]. Based on its neuroprotective and anti-inflammatory functions, PRG is considered a drug candidate for the treatment of various inflammatory and autoimmune diseases [21–24]. Treatment with PRG can reduce the signs of EAU in mice, decrease IL-17 levels, and increase IL-10 levels [22]. However, the effects of PRG on AU are unclear and the immunomodulatory mechanisms of PRG treatment need to be unraveled.

Unbiased high-throughput single-cell RNA sequencing (scRNA-seq) is a cutting-edge tool for anatomizing cellular heterogeneity and providing picturesque opportunities to gain novel insights into molecular mechanisms [11, 25]. Therefore, it would be desirable to establish a comprehensive atlas to understand the novel therapeutic mechanisms of PRG on AU treatment. Cervical draining lymph nodes (CDLNs) are the main draining site of eye and brain and facilitate the effective drainage of antigen and immune cells, where antigen-presenting cells and autoreactive Th17 react with the retinal antigens, and recruit inflammatory cells across the blood–eye barrier, thus causing local chorioretinal damage [26]. Removal of CDLNs can reduce disease severity of experimental autoimmune encephalomyelitis [27], suggesting the importance of CDLNs in autoimmune diseases. To this end, we constructed a comprehensive single-cell map of CDLNs in EAU mice treated with or without PRG, showing that PRG could reverse the imbalance of the immune cell composition in EAU-model mice, specifically increasing the number and function of Treg cells and decreasing the proportion and function of Th17 cells through the Id2/Pim1 pathway. Our work demonstrates the potential of PRG as a therapeutic agent for AU and refines our understanding of its protective mechanisms against autoimmune diseases.

## Materials and methods

### Mice

C57BL/6J mice (female, 6–8 weeks old, 20–25 g) were purchased from Guangzhou Animal Experiment Company. They were housed under specific pathogen-free conditions and maintained on a standard 12 h:12 h light–dark cycle with food and water provided ad libitum. All experiments were performed in compliance with the ARVO Animal Statement for the Use of Animals in Ophthalmic and Vision Research.

### Induction of EAU model

On day 0, we injected mice subcutaneously with a 1:1 mixture of 200 μg retinal antigen interphotoreceptor retinoid-binding protein 1–20 (IRBP_1–20_) (amino acid sequence: GPTHLFQPSLVLDMAKVLLD; GL Biochem, Shanghai, China) and complete Freund’s adjuvant (Difco, Detroit, MI, USA) containing 2.5 mg/mL of *Mycobacterium tuberculosis* strain H37Ra (BD Difco, San Jose, CA, USA). After immunization, 0.25 ug pertussis toxin (PTX) (List Biological Laboratories, Campbell, California, USA) dissolved in phosphate-buffered saline (PBS) was injected intraperitoneally on days 0 and 2.

### Treatment with progesterone

The progesterone (Sigma-Aldrich, St. Louis, MO, USA) was dissolved in 1% dimethyl sulfoxide (DMSO), 30% polyethylene glycol (PEG) 300, and 69% PBS solution. Then the EAU mice received intraperitoneal injection of progesterone (50 mg/kg) daily from day 2 until day 14 after EAU immunization. The control EAU mice were injected with the vehicle solution only. On day 14 after immunization, tissue samples were taken for subsequent histological studies, flow cytometry analysis and scRNA-seq.

### Clinical and histopathologic assessment

On day 14 after immunization, the fundus photography of the mice was performed with a Micron IV retinal imaging microscope (PHOENIX, USA). Clinical scoring (0–4 points) was performed based on previously published criteria, evaluated by retinal vasculitis, chorioretinal infiltration/lesions, papilledema, and retinal detachment [25]. For histopathologic examination, the eyeballs were extracted and immersed in 4% paraformaldehyde buffer for over 24 h, following by standard hematoxylin and eosin staining. Hematoxylin and eosin-stained eyeball sections exhibited cell infiltration and retinal folding with detachment. The histological and clinical severity was graded (0–4 points) referring to previously published criteria [25].

### Preparation of cell suspension of CDLNs

On day 14 after immunization, the CDLNs of naïve, EAU and PRG-treated EAU mice were extracted and the cell suspensions were prepared by grinding the organs through nylon mesh and then centrifuged at 1200 rpm for 5 min to get the cell pellet. Then, the cells were prepared for subsequent studies. For scRNA-seq in naïve, EAU and PRG-treated EAU mice, CDLNs cells from three mice in the same group were combined in one sample to ensure a sufficient number of cells for sequencing.

### Treatment of CDLNs cells in vitro

After isolation of CDLNs cells, the cells were seeded into 96-well plates (5 × 10^5^ cells/well) and treated with different stimulation conditions. Isolated CDLN cells were cultured with IRBP_1-20_ (20 μg/mL) alone or IRBP_1-20_ plus PRG (10 μM) with or without helichrysetin (HELI) (50 μM, TargetMol). In addition, cells were also cocultured with IL-23 (20 ng/mL, PeproTech, USA) or IL-23 plus PRG. The cells were stimulated at 37 °C and 5% CO_2_ environment for 72 h.

### Flow cytometry analysis

Cells were isolated from the retina and CDLNs on day 14 after immunization. Dead cells were excluded using live/dead dye (#423105, BioLegend, San Diego, CA, USA). Then they were stained with the following antibodies: the surface markers included: CD4 Percp-Cy5.5 (#100434), CD45 Brilliant Violet 605 (#103155) (BioLegend), TGFBR2 PE (#FAB532P, R&D Systems). For intracellular cytokine staining, the cells were stimulated with 5 ng/mL of phorbol myristate acetate, 500 ng/mL ionomycin, and 1 mg/mL brefeldin A (Sigma) at 37 °C for 5 h, following by fixation and permeabilization for 30 min. Then, cells were stained with antibodies detecting: IFN-γ PE (#505808), IL-17A Alexa Fluor 647 (#506912), FOXP3 FITC (#11-5773-82), Id2 PE-Cy7 (#25-9475-82, Invitrogen). For the Pim1 staining, cells were stained with surface antibodies, fixed, permeabilized, stained with Pim1 antibody (#3247S), then stained with Alexa Fluor 488-labeled antibody (#4412S) (Cell Signaling Technology, Danvers, USA). Finally, the cells were kept overnight at 4 ^◦^C and measured by flow cytometry. The flow cytometer (BD LSRFortessa, USA) was used for analysis and the results were analyzed with FlowJo software (version 10.0.7, Tree Star, Ashland, OR, USA).

### ELISA

The serum from mice of naïve, EAU and PRG group were determined using the mouse progesterone ELISA Kit (Elabscience, China) according to the manufacturer's instructions.

### Adoptive transfer experiment

The CD4 + lymphocytes of the EAU mice were separated on days 14 after immunization, and stimulated by IRBP_1–20_ (20 μg/mL) under Th17-polarizing conditions with and without progesterone for 3 days. Then the cells were injected into normal C57BL/6J mouse after being washed with PBS twice (2 × 10^7^ cells per mouse).

### scRNA-seq

#### scRNA-seq data alignment, processing, and sample aggregation

Using the Chromium Single Cell 5′ Library (10X Genomics, Genomics chromium platform Illumina NovaSeq 6000), Chip Kit (10X Genomics), Gel Bead and Multiplex Kit, the single-cell suspensions of CDLNs were transformed into barcoded scRNA-seq libraries. The quality of the libraries was checked by FastQC software. The CellRanger software (version 4.0; 10X Genomics) was applied to the preliminary processing of the Sequencing data. The count pipeline was used to demultiplex and barcode the sequences. Based on the single-cell expression matrix calculated by CellRanger, cells with fewer than 600 detected genes and a mitochondrial gene ratio greater than 15% were excluded. Finally, a total of 40,873 cells (naïve, 12,586 cells; EAU, 13,307 cells; PRG, 14,980 cells) were analyzed for the subsequent studies, including normalization, dimension reduction, clustering and differential gene expression analysis by using Seurat package (version 4.0.5) with the default parameters. The R package harmony was used to remove batch effect. The markers of each subset are listed in Additional file 2: Table S1.

#### Differential expression analysis

Before performing the differential expression analysis, the cell types that were missing or had fewer than three cells in the comparison groups were filtered out. Differential expression analysis for each cell type between different groups was performed by using the Wilcoxon rank-sum test implement in the ‘‘Find-Markers’’ function of the Seurat package (version 4.0.5). Similar with previous studies [28, 29], DEGs between the EAU and naïve groups were identified to generate an EAU-related DEG dataset (EAU-DEGs) (|LogFC|> 0.25, *P* value < 0.05). DEGs between the PRG and EAU groups were identified to establish a PRG-related DEG dataset (PRG-DEGs) (|LogFC|> 0.25, *P* value < 0.05). Based on the above results, ‘‘rescue-DEGs’’ were defined as the upregulated or downregulated EAU-DEGs that were downregulated or upregulated, respectively, by PRG treatment.

#### Gene functional annotation and enrichment analysis

The use of the Metascape web for GO biological process and pathway analysis enabled us to visualize the functional patterns of DEGs and perform statistical analysis. Finally, we used the heatmap and ggplot2 R package to visualized the top 10 of 30 terms or pathways that enriched by EAU-DEGs, PRG-DEGs or rescue-DEGs.

### Transcription factor–target gene network analysis

The transcriptional factors analysis was performed with the pyscenic (version 0.12.0) workflow using default parameters. Transcriptional factors (TFs) of mm9 which were used as reference TFs were downloaded from cistarget. The grn function was used to infer the co-expression network of TFs and target genes. Based on the ctx function on the mm9 database, TF-motif enrichment analysis was performed to identify the potential regulons and predict candidate target genes of TFs. After intersection of the predicted target genes of TFs and the downregulated rescue-DEGs of Th17 cells, “rescue target genes” of TFs were identified and the top 20 by importance was visualized using Cytoscape.

### Gene expression correlation analysis

The ggcorrplot R package was used to perform gene expression correlation analysis of downregulated rescue-DEGs of Th17 cells and the genes related to T cell activation, Th17 cell differentiation, IL-17 signaling pathway. Genes related to T cell activation, Th17 cell differentiation, IL-17 signaling pathway were obtained from Gene Ontology (GO:0042110), Th17 cell differentiation pathway SuperPath, and IL-17 Family Signaling Pathways SuperPath, respectively. The expression correlation analysis for each gene was performed by using the Spearman test implemented in the ggcorrplot package. Finally, we screened the genes that significantly correlated with *Id2*, with the correlation score > 0.15 and *P* value < 0.01 (Additional file 2: Table S4).

### Statistics and reproducibility

The two-tailed Student’s t-tests was used to compare the numerical variables between the EAU and PRG groups. The one-way ANOVA test was used to compare the numerical variables among the naïve, EAU, PRG groups in vivo, or the different conditions in vitro. Then the data analysis and presentation of verification experiments were performed using GraphPad Prism (version 8.0.2; GraphPad Software Inc., La Jolla, CA). In addition, the two-sided Wilcoxon test was used to compare the level of genes between EAU and naïve groups, or PRG and EAU groups. When calculating the GO biological process and pathway terms, *P* values were obtained through the hypergeometric test with the default parameters in Metascape webtool. Details of the size of biological replicates and the assays are provided in each of the figure legends. *P < 0.05, **P < 0.01, ***P < 0.001, ****P < 0.0001.

### Data availability

The scRNA-seq data are deposited in the Genome Sequence Archive in BIG Data Center, Beijing Institute of Genomics (BIG, https://bigd.big.ac.cn/gsa/), Chinese Academy of Sciences. The data of PRG mice were deposited under the GSA Accession No. CRA009938. The data of naïve and EAU mice were referenced to the young naïve and young EAU mice under the GSA Accession No. CRA004687. The data analysis pipeline used in scRNA-Seq follows the description on the 10X Genomics and Seurat official websites. The analysis steps, functions, and parameters used are described in detail in the Materials and methods section.

## Results

### PRG treatment attenuates EAU signs and alleviates intraocular inflammation

**Fig. 1:**
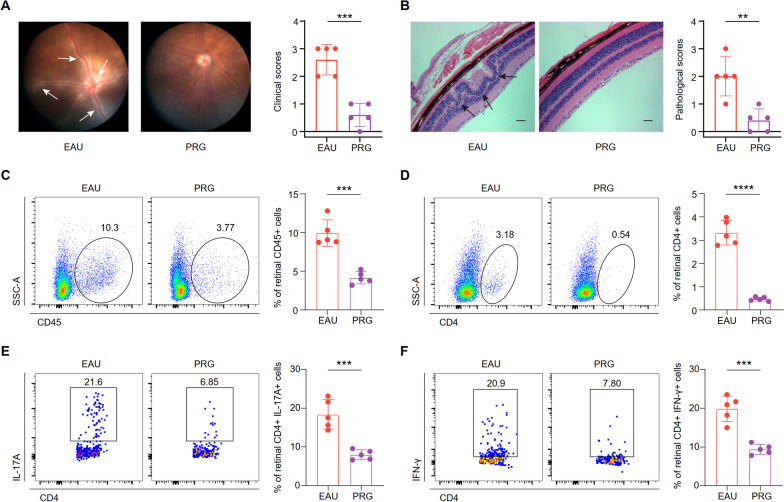
PRG treatment attenuated EAU symptoms and alleviated intraocular inflammation. **A** The representative fundus images of EAU and PRG groups (left). The white arrows indicate inflammatory exudation and linear lesions. The column charts showing the clinical scores between two groups (*n* = 5/group, right). **B** The representative H&E-stained images of EAU and PRG groups (left). Scale bars: 50 μm. The black arrows indicate retinal folding. The column charts showing the histological scores between two groups (*n* = 5/group, right). The flow cytometry histograms (left) and column charts (right) showing the percentage of CD45 + (**C**) and CD4 + cells (**D**) in retinal cells of two groups (*n* = 5/group). The flow cytometry histograms (left) and column charts (right) showing the percentage of CD4 + IL-17A + Th17 (**E**) and CD4 + IFN-γ + Th1 cells (**F**) in retinal cells of two groups (*n* = 5/group). Significance in **A**–**F** was calculated using two-tailed unpaired Student's t-test; **P < 0.01, ***P < 0.001, ****P < 0.0001

To determine whether PRG could ameliorate AU in vivo, we developed an EAU mouse model using interphotoreceptor retinal binding protein 1–20 (IRBP_1–20_), treated it with PRG (50 mg/kg) or vehicle daily, and evaluated the severity of EAU in two groups of mice. Multiple chorioretinal lesions and inflammatory cell infiltration were observed in EAU-model mice treated with vehicle, but not in the naïve mice (Fig. 1a, Additional file 1: Fig. S1a). Further, H&E staining images showed inflammatory infiltration and substantial retinal folding with detachment in the EAU mice (Fig. 1b, Additional file 1: Fig. S1b). However, PRG ameliorated these disease signs and dampened inflammation scores, both clinically and pathologically, in contrast to that observed in control EAU mice (Fig. 1a, b). These results indicate that PRG could effectively alleviate EAU in mice.

The infiltration of inflammatory cells (especially pathological T cells) into the eyes is crucial for pathological damage [30]. Therefore, we investigated whether PRG treatment could reduce intraocular inflammation. Flow cytometry was performed, and the proportions of retinal infiltrating CD45 + immune cells and CD4 + T cells in PRG-treated mice were much lower than those in control EAU mice (Fig. 1c, d, Additional file 1: Fig. S1c). Moreover, the levels of eye-infiltrating IL-17A + (Th17) and IFN-γ + (Th1) CD4 + T cells also showed a similar trend (Fig. 1e, f, Additional file 1: Fig. S1d). Therefore, PRG treatment protects mice from retinal injury after EAU induction by relieving EAU inflammation and reducing the penetration of effector CD4 + T cells into the retina.

### PRG treatment alters the immune cell profile in CDLNs of EAU

**Fig. 2:**
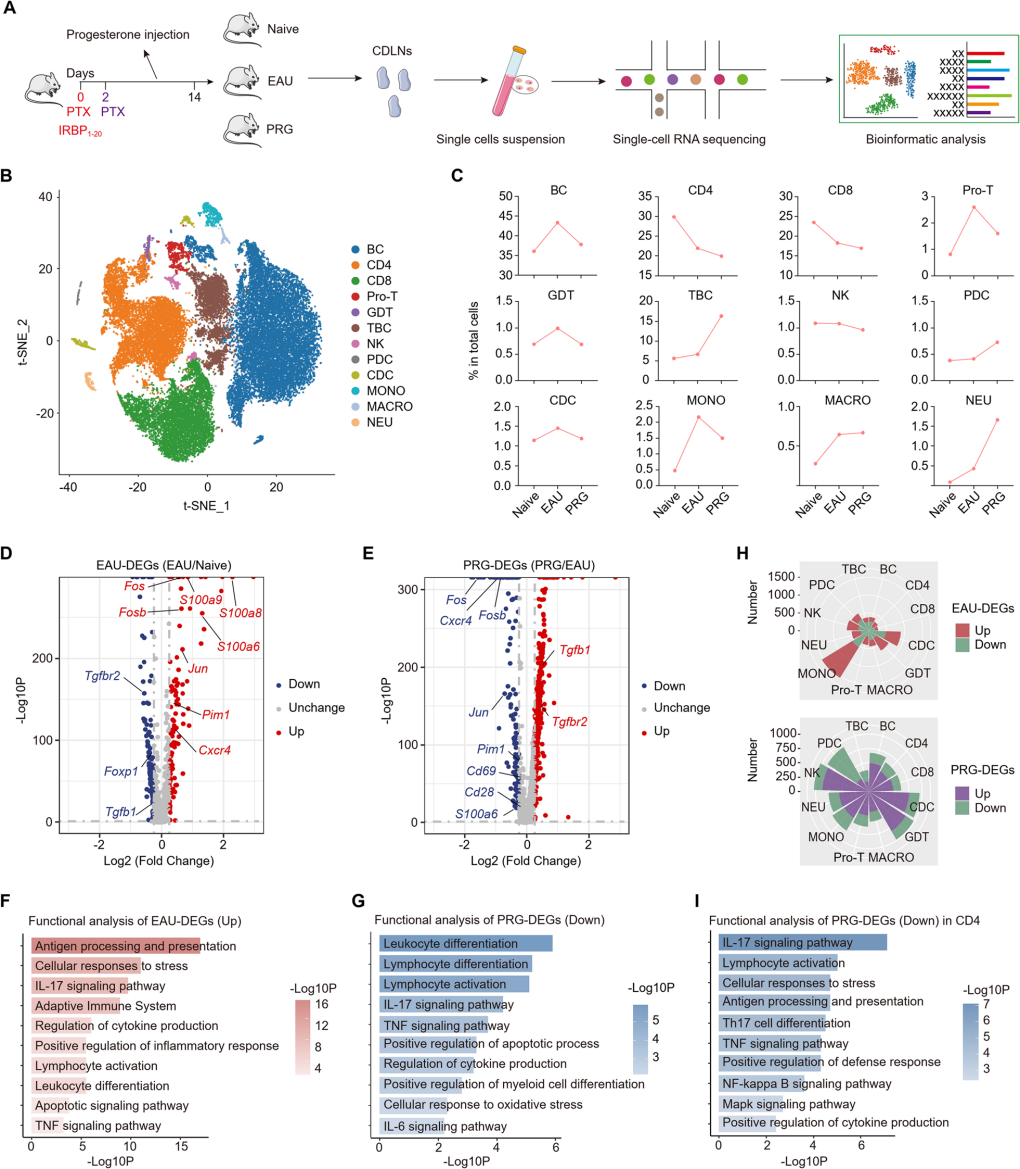
PRG treatment altered the immune cell profile of EAU. **A** Schematic diagram of experimental design for scRNA-seq analysis of CDLNs cells from Naïve group, EAU group and PRG group. **B** t-SNE plot showing the immune cell types of CDLNs in scRNA-seq. **C** Line charts showing the proportion of immune cell types, respectively, among three groups. **D** Volcano plot showing the EAU-DEGs in all cells. **E** Volcano plot showing the PRG-DEGs in all cells. **F** Representative GO biological process and pathways enriched in upregulated EAU-DEGs in all cells. **G** Representative GO biological process and pathways enriched in downregulated PRG-DEGs in all cells. **H** Rose diagrams showing the number of up and downregulated EAU-DEGs (up) or PRG-DEGs (down) among all immune cell types. **I** Representative GO biological process and pathways enriched in downregulated PRG-DEGs in CD4 + T cells

The activation of immune cells in CDLNs is essential for the recognition and presentation of intraocular antigens and for the onset of AU [31]. The size of CDLNs was expanded in EAU group and decreased after PRG treatment (Additional file 1: Fig. S2a). Therefore, to elucidate the potential molecular mechanisms underlying the therapeutic effects of PRG, immune cells were extracted from CDLNs of naïve, EAU-model, and PRG-treated EAU-model mice for scRNA-seq analysis (Fig. 2a). We obtained 40,873 cells after quality control (naïve, 12,586 cells; EAU-model, 13,307 cells; and PRG-treated EAU-model mice, 14,980 cells) for downstream analysis. Based on marker genes, we identified 12 major immune cell lineages as follows: BCs, CD4 + T cells (CD4), CD8 + T cells (CD8), proliferating T cells (Pro-T), gamma delta T cells (GDT), T and B cells (TBC), NK cells, plasmacytoid DCs (PDCs), conventional DCs (CDCs), monocytes (MONO), macrophages (MACRO), and neutrophils (NEU) (Fig. 2b, Additional file 1: Fig. S2b). We further found that T cells and BCs were dominant among CDLN cells, and the cellular ecosystem was reconstituted by EAU and PRG (Fig. 2c, Additional file 1: Fig. S2c).

To identify the molecular events associated with EAU and PRG treatment, we conducted differentially expressed gene (DEG) analysis between EAU-model and naïve mice (named EAU-DEGs) and between PRG-treated and EAU mice (named PRG-DEGs). During EAU, inflammatory genes (S100 family, AP-1 family) and autoimmune-related genes (*Pim1*, *Cxcr4*) were upregulated, whereas genes related to immunomodulation (*Tgfbr2* and *Foxp1*) were downregulated (Fig. 2d). Nevertheless, the trend during EAU was reversed following PRG treatment. In addition, PRG reduced the expression of genes related to cell activation (*Cd69*, *Cd28*) (Fig. 2e). Next, Gene Ontology (GO) and pathway analyses were performed to analyze the biological implications. The enriched processes related to cytokine production, cell activation, and the IL-17 pathway was notably upregulated during EAU, whereas it was downregulated by PRG treatment (Fig. 2f, g).

To separate the effects of EAU and PRG on each subset, we next analyzed EAU- and PRG-DEGs of each subpopulation. Myeloid subsets showed more DEGs than other cell types during EAU and PRG treatment (Fig. 2h). In MONO subset, EAU increased several genes related to myeloid cell activation and migration, like *Isg15*, *Il1b*, *Ccr2*, and *Csf1r*. PRG treatment reversed the upregulation of these genes (Additional file 1: Fig. S2d, e). In CD4, EAU increased the level of genes associated with T cells activation and Th17 differentiation, like *Rora*, *Pim1*, *Bhlhe40*, and *Hif1a* (Additional file 1: Fig. S2f). In addition to inhibiting these trends, PRG also increased several immunoregulatory genes, like *Tgfb1* and *Tgfbr2* (Additional file 1: Fig. S2g). GO and pathway analysis indicated that the PRG-downregulated genes in CD4 were enriched in the IL-17 signaling pathway, inflammatory pathways, and Th17 cell differentiation (Fig. 2i). We also performed DEGs analysis in BCs, the other important cells in AU pathogenesis. We found EAU elevated the genes related to B cell activation and antibodies production, like *Mzb1*, *Jchain*, *Ighg1*, *Pim1*, which were decreased after PRG treatment (Additional file 1: Fig. S2h, i). Finally, we explored the effects of PRG on NEU and TBC subsets. Although these two subsets were increased in PRG group, the genes related to inflammatory response and cell activation were decreased by PRG (Additional file 1: Fig. S2j, k). Collectively, PRG therapy inhibited several cell-specific inflammatory genes and pathways in EAU mice.

### PRG reverses EAU-induced PRG-related pathway disequilibrium

Next, we explored whether PRG-related pathways are altered in EAU-model mice. We analyzed the expression of genes related to PRG pathways in each subset and found that *Stat5b*, *Foxo1*, *Afp*, and *Cav1* were primarily expressed in CD4, whereas most genes were highly expressed in myeloid cells, such as those of the AP-1 family in NEUs (Additional file 1: Fig. S3a). In addition, several genes were altered in the EAU-model and PRG-treated EAU-model mice. The expression of *Fos*, *Fosb*, and *Ccl2* was increased in EAU-model mice and decreased after PRG treatment. Notably, the relatively low expression of immunomodulatory genes (*Prmt2*, *Foxo1*) was also reversed by PRG (Additional file 1: Fig. S3b). Protein arginine methyltransferase 2 (Prmt2) is a new member of the NF-κB pathway that controls LPS-induced inflammatory responses by regulating the nuclear accumulation of NF-κB and inhibiting the inflammation of vascular smooth muscle cells [32, 33]. Foxo1 mediates TGF-β-induced anti-inflammatory macrophage M2-like polarization [34]. The loss of Foxo1 in intestinal epithelial cells leads to an impaired gut microenvironment and enhanced susceptibility to intestinal inflammation [35]. Moreover, we verified the serum PRG levels of the three groups using ELISA, and the level was found to be decreased during EAU and increased after PRG treatment (Additional file 1: Fig. S3c). Collectively, EAU induced the disequilibrium of PRG and its associated pathways, whereas PRG treatment could recover this homeostasis.

### Reversal of EAU-induced changes in gene expression and inflammatory responses by PRG

**Fig. 3:**
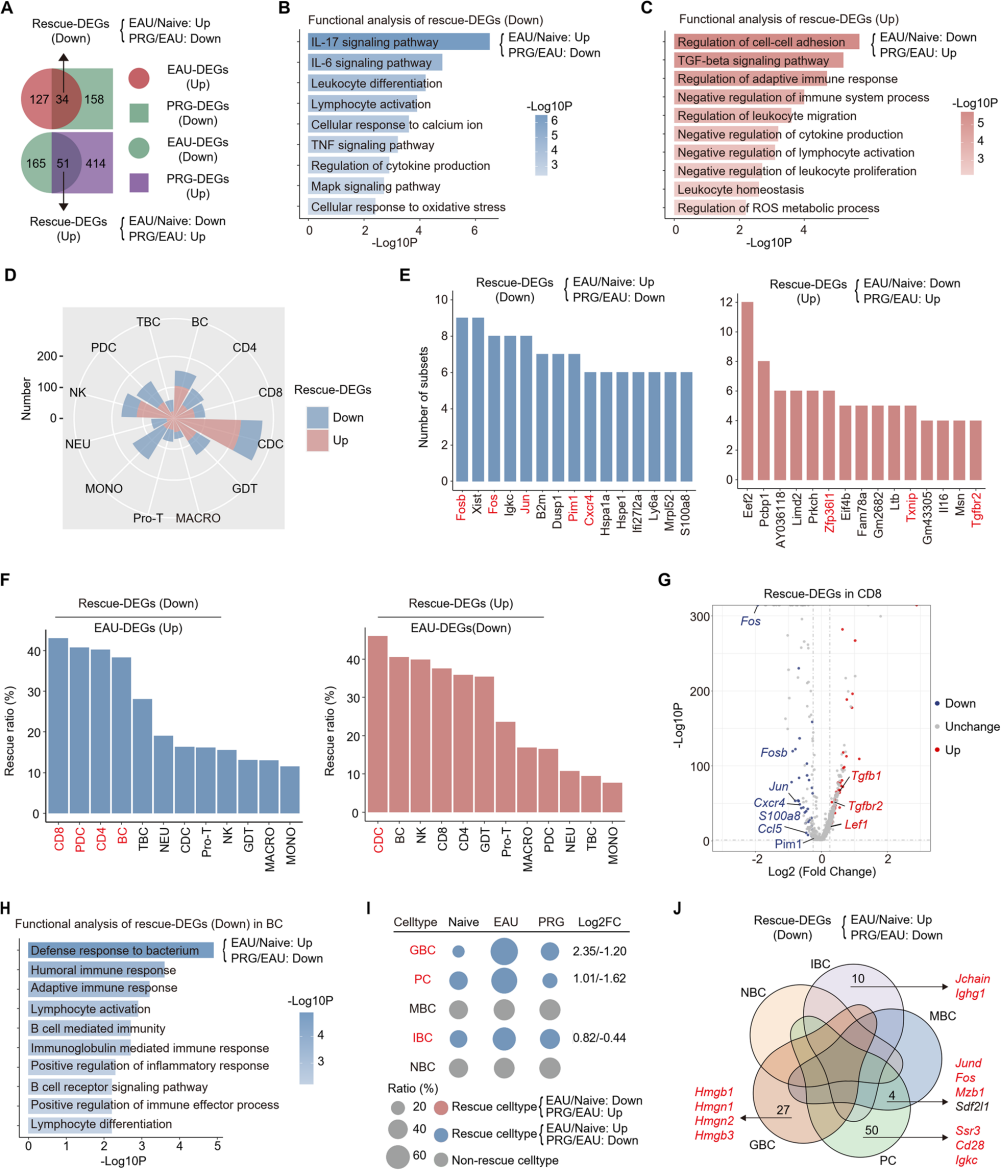
Reversal of EAU-induced changes in gene expression and inflammatory responses by PRG. **A** Venn diagram showing the interaction (up) of upregulated EAU-DEGs and downregulated PRG-DEGs and that (down) of downregulated EAU-DEGs and upregulated PRG-DEGs in all cells. The black arrows indicate down (up) or upregulated (down) rescue-DEGs. **B** Representative GO biological process and pathways enriched in downregulated rescue-DEGs in all cells. **C** Representative GO biological process and pathways enriched in upregulated rescue-DEGs in all cells. **D** Rose diagram showing the number of up and downregulated rescue-DEGs among all immune cell types. **E** Bar chart showing the frequencies of the top 15 rescue-DEGs observed across all immune cell types. **F** Bar chart showing the ratio of rescue-DEGs to EAU-DEGs in each immune cell type. **G** Volcano plot showing the up and downregulated rescue-DEGs in CD8 + T cells. **H** Representative GO biological process and pathways enriched in downregulated rescue-DEGs in B cells. **I** Dot plot showing the relative changes in cell ratios of BC subsets across the three groups. The numbers on the right indicate the Log2FC values of the cell ratios (EAU/Naïve and PRG/EAU). **J** Venn diagram showing the interaction of downregulated rescue-DEGs in BC subsets

Next, we explored the specific modulatory effects of PRG on EAU-induced immune changes. Integrative comparative analysis of EAU-DEGs and PRG-DEGs identified EAU-DEGs that were partially rescued by PRG, and these are referred to as ‘‘rescue-DEGs’’ (Fig. 3a). For example, upregulation of AP-1 family (*Fos*, *Jun*) and inflammatory genes (*Cxcr4*, *Ly6a*, *S100a13*, *Pim1*) during EAU was reversed by PRG, whereas downregulation of the naïve phenotype marker *Lef1* and regulatory gene *Tgfb1* was upregulated by PRG (Additional file 1: Fig. S3d). Enrichment analysis indicated that the genes upregulated during EAU were enriched in IL-17 and IL-6 pathways, cell differentiation, and activation, and their upregulation was reversed by PRG (Fig. 3b). In addition, the upregulated rescue-DEGs were enriched in the TGF-β pathway and in several processes related to immunoregulatory functions (Fig. 3c), suggesting that PRG could antagonize EAU-induced immunomodulatory dysfunction. Functional analysis of rescue-DEGs further supported the role of PRG in anti-inflammatory and immunomodulatory pathways.

Next, we explored the cell type-specificity of rescue-DEGs across cell subpopulations. As shown in the rose diagram, CDCs had the most rescue-DEGs (Fig. 3d). Notably, the levels of inflammatory and autoimmune genes (*Pim1*, *Cxcr4*, AP-1 family) were increased with EAU and rescued after PRG treatment in more than six cell subpopulations. In addition, levels of several immunomodulatory genes (*Zfp36l1*, *Txnip*, *Tgfbr2)* were decreased in more than four cell types from EAU-model mice and increased in those from PRG-treated mice (Fig. 3e). We further investigated the cell specificity of the rescue effects of PRG based on the ratio of rescue-DEGs to EAU-DEGs (Fig. 3f). CD8, PDCs, CD4, and BCs were the four cell types with the highest ratios of downregulated rescue-DEGs that were most effectively restored to a state of homeostasis by PRG. Expression of approximately 40% of the EAU-upregulated genes in these four types of cells was effectively reversed by PRG. In contrast, that in CDCs was rescued by PRG, which had the highest ratio of upregulated rescue-DEGs (Fig. 3f). Thus, we further analyzed the rescue-DEGs across these cell subpopulations. In CD8, EAU increased the levels of inflammatory genes and decreased the expression of immunoregulatory genes, which was reversed by PRG (Fig. 3g). In PDCs and CDCs, expression of the EAU-induced upregulated genes related to cell activation, the AP-1 family, and the interferon pathway was rescued by PRG (Additional file 1: Fig. S3e, f). Moreover, in CDCs, the inhibitory ligand-encoding gene *Icosl* was downregulated with EAU and upregulated after PRG treatment (Additional file 1: Fig. S3f). This result is consistent with the upregulation of *ICOSLG* during pregnancy in our previously published paper [11].

The other two cell subtypes with high ratios of downregulated rescue-DEGs, BCs and CD4, are important mediators of the autoimmune response and EAU development. GO and pathway analyses of downregulated rescue-DEGs in BCs indicated that the genes upregulated during EAU were enriched in the humoral immune response and BC differentiation and activation, and this increase in expression was suppressed by PRG (Fig. 3h). Next, we subdivided BCs into five subgroups, naïve B cells (NBCs), *Isg15* + naïve B cells (IBCs), memory B cells (MBCs), plasma cells (PCs), and germinal B cells (GBCs) (Additional file 1: Fig. S3g). PRG treatment reversed the EAU-induced accumulation of IBCs, PCs, and GBCs, which are the three key subsets involved in BC activation (Fig. 3i). We next attempted to determine whether canonical BC markers and inflammatory genes were regulated by EAU and PRG in a cell type-specific manner. As shown in the Venn plot, the effects of EAU and PRG were cell type-specific. In IBCs, the expression of genes related to the antibody response (*Jchain*, *Ighg1*) was increased with EAU and decreased after PRG treatment. Similar subtype-specific rescue patterns were also identified, including genes related to BC activation in PCs and the high-mobility group family in GBCs. Among the four genes shared by MBCs and PCs, expression of those related to the AP-1 family and antibody response (*Mzb1*) was increased in the EAU group and decreased in the PRG group (Fig. 3j). Taken together, these data revealed that PRG treatment reprogramed EAU-compromised gene expression and inflammatory responses. These EAU-DEGs across cell types of which expression was commonly rescued by PRG might represent important biomarkers and targets for PRG intervention.

### PRG modulates the Th17/Treg imbalance and upregulates frequency and functional molecules expression of Treg

**Fig. 4:**
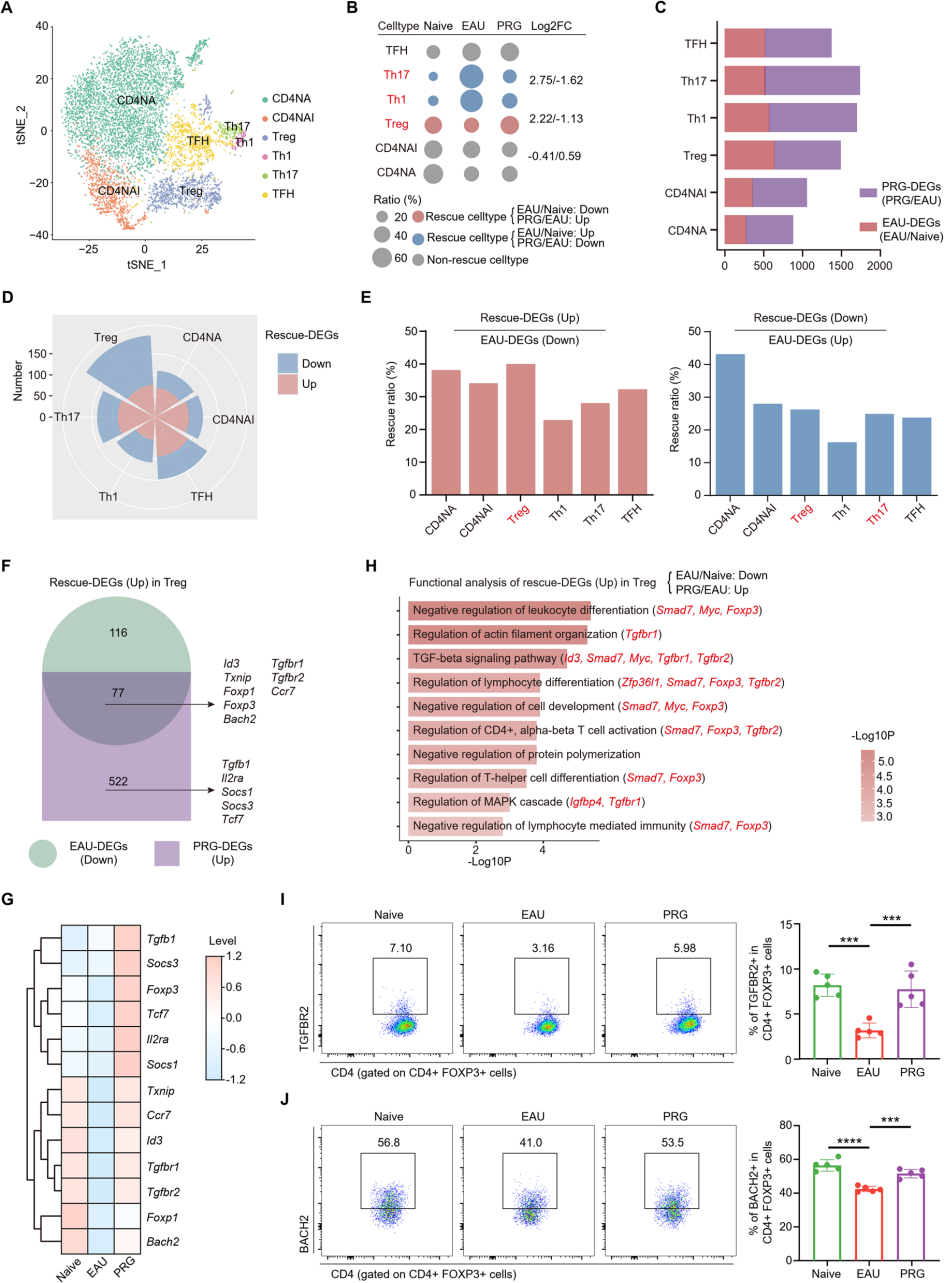
PRG modulated Th17/Treg imbalance and promoted the frequency and function of Treg. **A** t-SNE plot showing the CD4 + TC subsets of CDLNs in scRNA-seq. **B** Dot plot showing the relative changes in cell ratios of CD4 + T cell subsets across the three groups. The numbers on the right indicate the Log2FC values of the cell ratios (EAU/Naive and PRG/EAU). **C** Bar chart showing the number of PRG-DEGs and EAU-DEGs in CD4 + TC subsets. **D** Rose diagram showing the number of up and downregulated rescue-DEGs in CD4 + TC subsets. **E** Bar chart showing the ratio of rescue-DEGs to EAU-DEGs in each CD4 + TC subset. **F** Venn diagram showing the interaction of downregulated EAU-DEGs and upregulated PRG-DEGs in Treg cells. **G** The heatmap showing the relative levels of specific genes among three groups. **H** Representative GO biological process and pathways enriched in upregulated rescue-DEGs in Treg cells. The flow cytometry histograms (left) and column charts (right) showing the level of TGFBR2 (**I**) and BACH2 (**J**) in CD4 + FOXP3 + Treg cells among three groups (*n* = 5/group). Significance in **I**, **J** was calculated using one-way ANOVA test; ***P < 0.001, ****P < 0.0001

CD4 + T cells activation and the Th17/Treg imbalance play important roles in EAU pathogenesis. In our study, expression of nearly 40% of the EAU-upregulated genes was rescued by PRG treatment (Fig. 3f), suggesting that PRG might have regulatory effects on EAU-induced CD4 + T cells activation. Indeed, GO and pathway analyses indicated that PRG could inhibit several EAU-induced autoimmune-related pathways and processes in CD4 + T cells, such as the IL-17 pathway, Th17 differentiation, lymphocyte activation, and cytokine production (Additional file 1: Fig. S4a). To explore the influence of PRG on CD4 + subsets, we divided these cells into six subgroups (Fig. 4a, Additional file 1: Fig. S4b). Among these, PRG reversed the EAU-induced increase in Th17 and Th1 cell subsets and reversed the decrease in Treg cells (Fig. 4b). When analyzing the EAU-DEGs and PRG-DEGs in these subsets, we found that the effect of PRG on the EAU group was more obvious than the effect of EAU on the naïve group (Fig. 4c). In addition, the rose diagram showed that the Treg subgroup had the most rescue-DEGs (Fig. 4d, Additional file 1: Fig. S4c). Moreover, Tregs had the highest ratio of upregulated rescue-DEGs, suggesting that the expression of approximately 40% of the downregulated EAU-DEGs was increased by PRG (Fig. 4e). In terms of the downregulatory effects of PRG, Treg and Th17 were the top two cell types most strongly affected by EAU and PRG, and these were more effectively rescued by PRG treatment among memory and effector CD4 + subsets (Fig. 4e). These results indicate that PRG could reverse CD4 + T cells activation and the Th17/Treg imbalance during EAU.

Treg is the key agent that maintains the immune homeostasis. EAU decreased the Tregs frequency and this downregulation could be restored to a normal level by PRG (Additional file 1: Fig. S4d, e). Next, we conducted a detailed analysis of the Treg subgroup and found that the expression of several genes encoding transcription factors (TFs, including *Id3, Txnip*, *Foxp1*, *Foxp3*, *Bach2*) and receptors (*Tgfbr1, Tgfbr2, Ccr7*) was decreased with EAU, but reversed by PRG. In addition, we found that several genes (*Tgfb1*, *Il2ra*, *Tcf7*, *Socs1*, *Socs3*) were upregulated by PRG compared to EAU (Fig. 4f, g, Additional file 2: Table S2). These genes were closely linked to the differentiation and immunoregulatory functions of Tregs [36–38]. Subsequent functional enrichment analysis was conducted on the upregulated rescue-DEGs in Tregs, which showed that the pathways related to TGF-β signaling and the negative regulation of leukocyte differentiation were enriched (Fig. 4h). Among these rescue-DEGs in Treg, the receptors Tgfbr1 and Tgfbr2 form a heterodimer and bind to TGF-β, which plays a crucial role in the differentiation and function of Tregs [39]. BACH2 is required for the survival and maintenance of resting Tregs, promoting immune homeostasis [40]. Consistent with the scRNA-seq findings, flow cytometry indicated that EAU downregulated the expression of TGFBR2 and BACH2 on Tregs, and these downregulations were reversed by PRG (Fig. 4i, j, Additional file 1: Fig. S4f, g). Similar trend was also observed in the IL-10 production of Treg (Additional file 1: Fig. S4h).

Collectively, PRG treatment increases the frequency and the expression of related functional molecules of Treg, and improves the balance of Th17/Treg cells, which might be an important mechanism through which PRG reduces EAU inflammation.

### Reversal of the EAU-induced increase in Th17 cell proportions and functional factors by PRG

**Fig. 5:**
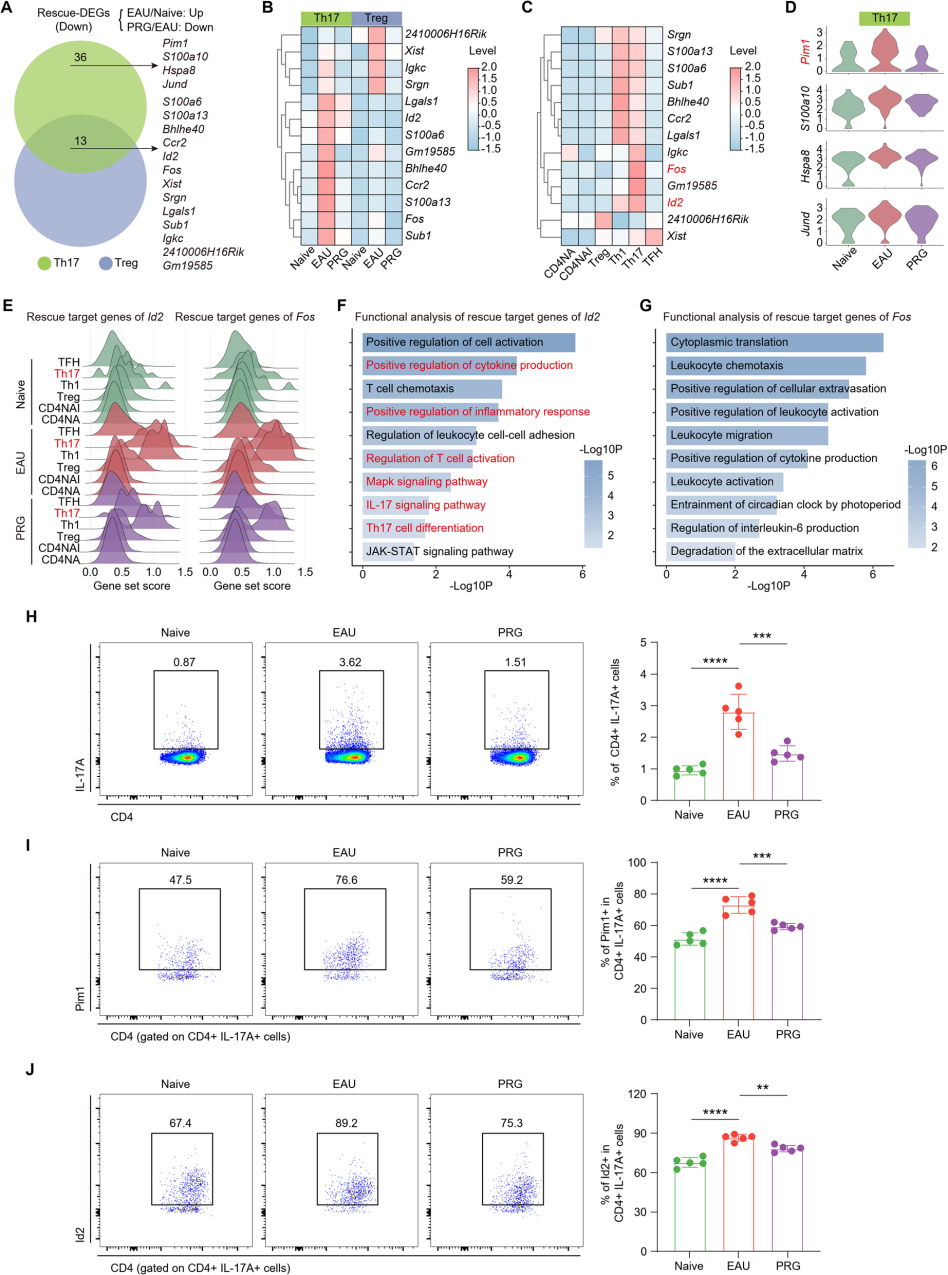
Inversion of EAU-induced increase in the proportion and functional genes of Th17 by PRG. **A** Venn diagram showing the interaction of Th17 and Treg downregulated rescue-DEGs. **B** The heatmap showing the relative levels of 13 genes in Th17 and Treg among three groups. **C** The heatmap showing the relative levels of 13 genes among CD4 + TC subsets. **D** Violin plot showing the expression of selected genes in Th17 cells among three groups. **E** Ridge plots showing the gene set scores of the “rescue target genes” of *Id2* (left) and *Fos* (right) in CD4 + TC subsets among three groups. **F** Representative GO biological process and pathways enriched in “rescue target genes” of *Id2*. **G** Representative GO biological process and pathways enriched in “rescue target genes” of *Fos*. The flow cytometry histograms (left) and column charts (right) showing the percentage of CD4 + IL-17A + Th17 cells (**H**), and the level of Pim1 (**I**) and Id2 (**J**) in Th17 cells among three groups (*n* = 5/group). Significance in **H**–**J** was calculated using one-way ANOVA test; **P < 0.01, ***P < 0.001, ****P < 0.0001

Th17 cells are the major CD4 + T cell subset that play key roles in EAU development. Among the memory and effector CD4 + subgroups, PRG had the most significant effect on reversing the upregulation of EAU-DEGs in Th17 and Treg cells (Fig. 4e). We then performed functional enrichment analysis on the downregulated rescue-DEGs in Th17 cells and found that they were mainly enriched in pathways related to cytokine production, T cell activation, the IL-17 signaling pathway, and Th17 cell differentiation (Additional file 1: Fig. S5a). Among the 49 downregulated rescue-DEGs in Th17 cells, 13 were also present in Tregs exhibiting the same trend in which expression was increased in EAU-model and decreased in PRG-treated mice, including the key mediators (*Bhlhe40* and *Ccr2*), involved in Th17 functions (Fig. 5a, b, Additional file 2: Table S3). These genes were highly expressed in memory and effector cells, especially Th17 and Th1 cells. We also identified several genes highly expressed in Th17 cells, including *Id2*, which mediates the CD4 + T cells immune response, and *Fos*, which plays a crucial role in autoimmunity and inflammation [41, 42] (Fig. 5c). Interestingly, the rescue effects of PRG on *Id2* were only present in Treg and Th17 cells (Additional file 1: Fig. S5b). In addition, we found that PRG reversed the elevated levels of inflammation- and autoimmune-related genes *(Pim1*, *S100a10, Hsap8*, *Jund*) in Th17 cells of EAU-model mice (Fig. 5a, d, Additional file 2: Table S3). We previously reported that Pim1 is closely linked to Th17 cell differentiation and pathogenicity [43]. These results suggest that EAU-mediated upregulated genes and processes are related to Th17 functions, which could be reversed by PRG treatment.

*Id2* and *Fos* play important roles in the immune response by encoding TFs. Accordingly, the “rescue target genes” encoding TFs in our data were identified by taking the intersections of TF target genes in the database and the downregulated rescue-DEGs in Th17 cells (Additional file 1: Fig. S5c). Genes downstream of *Id2* and *Fos* were upregulated in Th17 cells of the EAU group, which were suppressed by PRG (Fig. 5e). Notably, the genes downstream of *Id2* were determined to be involved in the regulation of the inflammatory response, the IL-17 signaling pathway, and Th17 cell differentiation, whereas genes downstream of *Fos* were not enriched in specific Th17-related pathways (Fig. 5f, g). GO enrichment analysis also showed that the *Id2* gene itself was involved in the pathway related to leukocyte differentiation and activation (Additional file 1: Fig. S5d). These results indicate that *Id2* might play an important role in Th17 cell differentiation and functions and serve as a key target for PRG intervention. Consistent with these findings, the high frequency of Th17 cells in EAU-model mice was decreased by PRG (Fig. 5h, Additional file 1: Fig. S5e). Notably, we also detected the same trends for Pim1 and Id2 in Th17 cells (Fig. 5i, j, Additional file 1: Fig. 5f, g). Taken together, our data suggest that PRG treatment increases the levels of functional molecules, such as Pim1 and Id2, and resets the regulatory programs that control Th17 differentiation and pathogenicity.

### PRG attenuates Th17 cell differentiation and pathogenicity during EAU through the Id2/Pim1 pathway

**Fig. 6:**
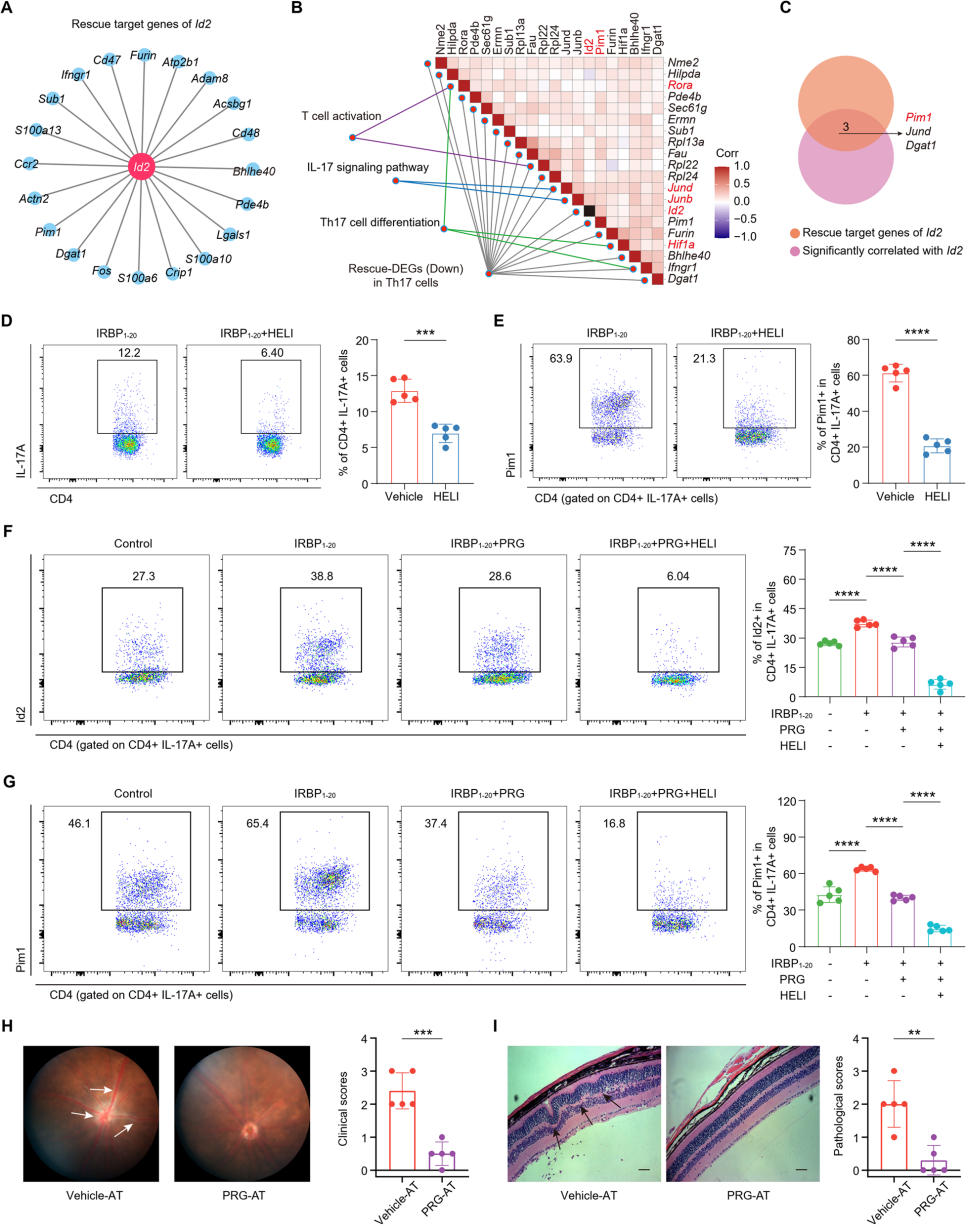
PRG attenuated Th17 differentiation and pathogenicity in EAU through Id2/Pim1 pathway. **A** Network plot showing the top 20 “rescue target genes” of *Id2* by importance. **B** The heatmap showing the expression correlation of downregulated rescue-DEGs of Th17 cells and these genes related to T cell activation, Th17 cell differentiation, IL-17 signaling pathway in the *Id2* module. **C** Venn diagram showing the screening of “rescue target genes” that significantly correlated with the level of *Id2*. The flow cytometry histograms (left) and column charts (right) showing the percentage of CD4 + IL-17A + Th17 cells (**D**) and the level of Pim1 + in Th17 cells (**E**) between IRBP_1-20_ or IRBP_1-20_ + HELI groups (*n* = 5/group). The flow cytometry histograms (left) and column charts (right) showing the level of Id2 (**F**) and Pim1 (**G**) in Th17 cells among control, IRBP_1-20_, IRBP_1-20_ + PRG and IRBP_1-20_ + PRG + HELI groups (*n* = 5/group). **H** The representative fundus images of Vehicle-AT and PRG-AT groups (left). The white arrows indicate inflammatory exudation and linear lesions. The column charts showing the clinical scores between two groups (*n* = 5/group, right). **I** The representative H&E-stained images of Vehicle-AT and PRG-AT groups (left). Scale bars: 50 μm. The black arrows indicate retinal folding. The column charts showing the histological scores between two groups (*n* = 5/group, right). Significance in **D**, **E** and **H**, **I** was calculated using two-tailed unpaired Student's t-test; significance in **F**, **G** was calculated using one-way ANOVA test; **P < 0.01, ***P < 0.001, ****P < 0.0001

Based on our previous findings, we hypothesized that Id2 and its target genes play an important role in the differentiation and function of Th17 cells. We took the intersections of the target genes of *Id2* in the database and the downregulated rescue-DEGs of Th17 cells and identified the top 20 “rescue target genes” by importance (Fig. 6a). To screen and identify the specific downstream genes that play a role in EAU development, we constructed *Id2*-related expression correlation maps. We then conducted expression correlation analysis of downregulated rescue-DEGs of Th17 cells and genes related to T cell activation, Th17 cell differentiation, and the IL-17 signaling pathway. We found that in the correlation module involving *Id2*, *Rora*, and *Hif1a* was closely related to Th17 cell differentiation and that *Junb* and *Jund* were closely related to the IL-17 pathway (Fig. 6b, Additional file 1: Fig. S6a). Moreover, among the “rescue target genes” of *Id2*, *Pim1* was most significantly correlated with *Id2* (Fig. 6b, c, Additional file 1: Table S4). These results suggest that Id2 plays an important promoting role in Th17 cells through Pim1.

To verify this hypothesis, we conducted in vitro experiments by using IRBP_1–20_ as a stimulator. We isolated CDLN cells from EAU-model mice and treated them with IRBP_1–20_ or IRBP_1–20_ plus helichrysetin (HELI), an inhibitor of Id2 [44]. We confirmed that HELI decreased the expression of Id2 both in CD4 + and Th17 cells (Additional file 1: Fig. S6b, c), indicating the inhibitory effect of HELI on Id2 expression. We next evaluated the effects of Id2 inhibition on Th17 cell frequency and found that the high frequency was decreased by HELI treatment (Fig. 6d). Notably, the Th17-derived expression of Pim1 also significantly decreased after addition of the Id2 inhibitor HELI (Fig. 6e). Therefore, the abnormal Id2/Pim1 pathway in EAU might contribute to Th17 cell differentiation and pathogenicity in uveitis, which could be ameliorated by Id2 inhibition.

Next, we explored whether PRG modulates the Id2/Pim1 pathway to attenuate Th17 cell functions. Consistent with the results of in vivo experiments, PRG treatment suppressed IRBP_1–20_-induced Th17 cell differentiation, which was enhanced by adding an Id2 inhibitor (Additional file 1: Fig. S6d). Similarly, the levels of Id2 and Pim1 in Th17 cells were assessed. IRBP_1–20_ treatment increased the proportion of Id2 + and Pim1 + Th17 cells. These IRBP_1–20_-induced effects were reversed by PRG treatment, and this suppression was enhanced upon co-culture with PRG and HELI (Fig. 6f, g). In addition, GM-CSF is a marker that distinguishes pathogenic Th17 cells from non-pathogenic Th17 cells [45]. Under the stimulation of IL-23, the receptor IL-23R on the surface of CD4 + T cells transmits signals to promote Th17 cells differentiation and Th17's secretion of IL-17, GM-CSF and other pathogenic cytokines. Targeting GM-CSF inhibits Th17 cell pathogenicity and attenuates EAU severity [46]. Flow cytometry indicated that the proportion of IL-23R + and GM-CSF + Th17 cells increased by addition of IL-23, and decreased after PRG treatment (Additional file 1: Fig. S6e, f). These results indicate that PRG could inhibit the pathological Id2/Pim1 axis and IL-23/Th17/GM-CSF signaling to suppress Th17 cell functions and AU progression.

The transfer of autoreactive IRBP_1-20_-specific T cells can induce EAU development [43]. To further confirm the inhibitory effects of PRG on the pathogenicity of IRBP_1-20_-specific T cells, we performed an adoptive transfer (AT) experiment. CDLNs cells of EAU-model mice were stimulated with IRBP_1-20_ along with the addition of PRG (PRG-AT) or vehicle (vehicle-AT) for 3 days. IRBP_1-20_-specific T cells induced EAU, whereas PRG-pretreated cells failed to induce EAU (Fig. 6h, i). Correspondingly, the amount of Id2 + and Pim1 + Th17 cells were decreased in PRG-AT mice compared to that in vehicle-AT mice (Additional file 1: Fig. S6g, h). In summary, we found that Id2 regulates Pim1 expression and Th17 cell functions during EAU development. Treatment with PRG can inhibit Th17 cell differentiation and pathogenicity by suppressing pathological Id2/Pim1 axis and GM-CSF signaling, further regulating the Th17/Treg imbalance and leading to the treatment of AU.

## Discussion

Here, based on the scRNA-seq analysis of CDLN cells from EAU-model mice undergoing PRG treatment, we systematically evaluated the effects of PRG treatment on EAU according to cell-type composition and genetic expression, which shows the therapeutic potential of PRG for EAU and provides novel insights into PRG regimens for AU treatment. The primary findings were summarized as follows: (1) PRG treatment remarkably ameliorated retinal lesions and inflammatory cell infiltration in EAU-model mice; (2) PRG reversed EAU-induced PRG-related pathway disruption; (3) PRG reversed EAU-induced gene expression and autoimmune processes related to cell differentiation, cell activation, and cytokine signaling pathways; (4) the EAU-induced BC activation and humoral immune response were rescued by PRG treatment; (5) PRG increased the number of and regulatory functional molecules of Tregs and orchestrates Th17/Treg disequilibrium; and (6) Id2 regulated the expression of Pim1 and promoted Th17 pathogenicity, which could be reversed by PRG to alleviate EAU inflammation and treat AU.

As a cholesterol-derived hormone, PRG is vital for the maintenance of normal functions during the menstrual cycle and in mammalian reproduction. PRG and novel PRG-based drugs can be used for the treatment of contraception, nerve injuries, inflammatory diseases, and cancer [14, 17, 47]. Combined with the unique maternal immune environment, the correlation between PRG levels and disease states suggests that PRG plays a role in the control of inflammatory diseases [10, 11, 48]. However, its potential therapeutic roles and mechanisms in autoimmune diseases remain unclear. Therefore, we used a commonly used mice model and scRNA-seq to explore the effects of PRG treatment on EAU and the underlying mechanisms. We found that PRG could reduce EAU disease signs and immune cell infiltration in the retina. The conflict with the results of previous published study [49] may be attributed to the differences in the used animal, drug-delivery way, and PRG dosage. High concentrations of PRG have been reported to exert immunomodulatory functions that inhibit the autoimmune response, while low concentrations may have the opposite effect [50]. Administration of high level of PRG could match the physiological serum levels of PRG in pregnant mice, thereby rescuing letrozole-induced implantation failure [51, 52]. The high levels of PRG may be the important reason for the therapeutic effects of PRG on EAU in this study. Similarly, we found that inflammation during EAU was apparently rescued by PRG, mainly manifested by the downregulation of autoreactive genes (such as *Pim1* and *Cxcr4*) and processes associated with cytokine pathways, leukocyte differentiation and activation. We have reported that Pim1 plays an important role in the pathogenesis EAU, suggesting that it could be a therapeutic target [25]. In addition, lymphocyte subsets, such as CD8, CD4, and BCs, were found most effectively restored from a state of overactivation with EAU to a state of homeostasis upon PRG treatment. The EAU-induced accumulation of key subsets involved in BC activation and signaling, upregulation of humoral immune response and BC differentiation and activation, were rescued by PRG. These results are consistent with the reduction of BC activation during pregnancy [11]. PRG has been reported to inhibit the antigen-presenting function of BCs to facilitate the establishment of fertilization and pregnancy [53]. Moreover, in the subgroup analysis of CD4 + cells, EAU-induced increase in Th17 and Th1 cells and the decrease in Treg cells were reversed by PRG. Although the frequency of NEU and TBC subsets were increased after PRG treatment, the genes related to inflammatory response and cell activation were decreased by PRG. The increase of NEU may be related to the inhibition of neutrophil apoptosis by PRG [54]. In conclusion, our study demonstrated the therapeutic effects of PRG on EAU and expanded our understanding of the immunomodulatory function of PRG.

CD4 + Treg cells are functionally distinct T subpopulations that potentiate immunological self-tolerance and immune homeostasis [19]. Abnormal Tregs, whether functional or quantitative, can lead to the occurrence of AD and other immune-related diseases [55]. The debilitation of adaptive immunity and aggrandized function of immune regulatory cells (such as Tregs) play vital roles during pregnancy. PRG drives the development of Treg cells through the receptor activator of NF-κB ligand in pregnant mice [56]. However, the effects of PRG on Tregs under autoimmune conditions remain unclear. In this study, Treg was the subpopulation most effectively restored to a regulatory state upon PRG treatment among CD4 + subsets, both in frequency and functional molecules level. Moreover, expression of several genes (*Tgfbr2, Bach2* and *Myc*) and the TGF-β pathway, which are important for the regulatory functions of Tregs, were decreased in EAU mice and upregulated by PRG treatment. The cell homeostasis and suppressive functions of Tregs are dependent on the TGF-β signaling pathway mediated by the binding of TGF-β to its receptors, Tgfbr1 and Tgfbr2 [57]. *Tgfbr2* deficiency in mice can cause lethal inflammation [39]. The transcriptional regulator c-Myc (Myc) orchestrates immune homeostasis by coordinating Treg accumulation, functional activation, and metabolic programming [58]. We also validated that PRG reversed EAU-induced decrease of functional molecules TGFBR2, BACH2 and IL-10 expression in Tregs. The upregulation of these mediators induced by PRG demonstrated the specific modulatory effects of PRG on Tregs, ultimately restoring the Th17/Treg balance. In addition, we found that the expression of the gene encoding the inhibitory ligand *Icosl* was decreased in DCs of EAU-model mice and reversed by the pregnancy level of PRG, which was consistent with the upregulation of ICOS–ICOS ligand in pregnant women [11]. The ICOS signaling is important for the establishment of tolerogenic immune responses by promoting the generation and functions of Tregs [59, 60]. Overall, these findings highlight the modulatory effects of PRG on the frequency and functional molecules levels of Tregs, thus leading to the treatment of AU.

Th17 cells are a pathogenic subset that promotes tissue inflammation and damage during AD pathogenesis [61]. Inhibiting Th17 cells, especially pathological Th17 cells, can restore the immune balance and lead to treatment of EAU and AU [25]. The downregulation of Th17 cell functions is critical for successful pregnancy, and miscarriage is often accompanied by their overactivation [62]. The level of PRG during pregnancy could overcome miscarriage disorders through the inhibition of Th17 cells [63]. However, the specific mechanisms through which PRG regulates Th17 cell differentiation and pathogenicity during AD have not been elucidated. We found the expansion of Th17 cells during EAU was inhibited by PRG, both in vivo and in vitro. Downregulation of the expression of the Th17 cell differentiation-related transcription factor RORγt could be the underlying mechanism [64]. Moreover, PRG specifically inhibited several important mediators (such as Pim1) and inflammatory pathways involved in Th17 cell pathogenicity. The inhibition of Pim1 could reduce CD4 + T cells activation and Th17 cell pathogenicity in patients with RA and Vogt–Koyanagi–Harada disease [25, 65]. Moreover, we identified that the novel molecule Id2 was critical for Th17 cell pathogenicity because it was highly expressed in Th17 cells, and the downstream target genes of Id2 were enriched in the IL-17 signaling pathway and Th17 cell differentiation. Id2 is required for the CD4 + T cells immune response, and its conditional knockout reduces T cell activation [41]. Acted as an inhibitor of E protein transcription factors, Id2 regulates the transcriptional activation of other molecules [66]. Therefore, we further indicated that *Pim1* was the candidate target gene of Id2 and was positively correlated with the level of Id2. Indeed, we validated that inhibiting Id2 could decrease Pim1 expression and Th17 cells frequency in vitro, suggesting that Id2 regulates Pim1 expression and Id2/Pim1 pathway is involved in the differentiation and pathogenicity of Th17 cells. Notably, after PRG treatment, the stimulus-induced activation of the Id2/Pim1 pathway was downregulated in Th17 cells, resulting in impaired Th17 cell activation and AU treatment. These results indicate that Id2 is a potential therapeutic target for EAU and AU, providing a basis for future study on targeted therapy. In addition, we validated that PRG also had modulatory effects on the pathogenic IL-23/Th17/GM-CSF pathway. Finally, our adoptive transfer experiments also showed that the transfer of CD4 + T cells treated with PRG under Th17-polarizing conditions failed to induce EAU. Collectively, the Id2/Pim1 pathway is critical for Th17 cell pathogenicity and EAU development, and PRG suppresses these processes, expanding the therapeutic potential of PRG.

## Conclusions

In summary, we elucidate the therapeutic effects of PRG on EAU and delineate the potential molecular mechanisms using a single-cell tool. We confirmed the potential of PRG to alleviate EAU inflammation by inhibiting inflammatory pathways and restoring the Th17/Treg balance. We also broaden our understanding of the regulatory effects of PRG on Treg functions under autoimmune conditions and the underlying mechanisms. Moreover, we identify Id2/Pim1 as an important pathway for Th17 cell pathogenicity during EAU, whereby PRG treatment decreases the activation of the Id2/Pim1 pathway and Th17 cell differentiation. Therefore, we demonstrate that PRG may be a new therapeutic alternative for ADs, and it has anti-autoimmune functions, not just pregnancy maintenance and neuroprotection. This study broadens our understanding of the immunomodulatory mechanisms underlying PRG treatment of AU and other ADs.

## Supplementary Information

**Additional file 1: Fig. S1.** The effects of PRG on retinal cells during EAU. **Fig. S2.** The clustering strategies for scRNA-seq of CDLNs cells. **Fig. S3.** PRG reversed the EAU-induced inflammatory responses and PRG-related pathway disequilibrium. **Fig. S4.** The modulatory effects of PRG on CD4+ T cells. **Fig. S5.** The modulatory effects of PRG on Th17 cells. **Fig. S6.** The modulatory effects of PRG on Id2/Pim1 pathway in Th17 cells.

**Additional file 2: Table S1.** Cell clustering strategy in scRNA-seq. **Table S2.** The differential expression analysis in Treg of Figure 4F-G. **Table S3.** The differential expression analysis in Th17 and Treg of Figure 5A. **Table S4.** The expression correlation analysis of downregulated rescue-DEGs of Th17 cells and the genes related to T cell activation, Th17 cell differentiation, IL-17 signaling pathway.

## Data Availability

The data are available from the corresponding author on reasonable request.

## Abbreviations

AD: Autoimmune diseases
AU: Autoimmune uveitis
BCs: B cells
CDLNs: Cervical draining lymph nodes
CD4: CD4 + T cells
CD8: CD8 + T cells
CDCs: Conventional dendritic cells
DCs: Dendritic cells
DEG: Differentially expressed gene
EAU: Experimental autoimmune uveitis
EAU-DEGs: Differentially expressed genes between EAU and naïve mice
GBCs: Germinal B cells
HELI: Helichrysetin
IBCs: *Isg15* + Naïve B cells
IRBP_1–20_: Interphotoreceptor retinal binding protein 1–20
MBCs: Memory B cells
MS: Multiple sclerosis
NEU: Neutrophils
NK: Natural killer cells
PCs: Plasma cells
PDCs: Plasmacytoid dendritic cells
PRG: Progesterone
PRG-DEGs: Differentially expressed genes between PRG-treated and EAU mice
scRNA-seq: Single-cell RNA sequencing
TF: Transcription factor
Th17: CD4 + T helper 17
Treg: CD4 + regulatory T cell
