# Correction: Progesterone attenuates Th17-cell pathogenicity in autoimmune uveitis via Id2/Pim1 axis

**DOI:** 10.1186/s12974-025-03412-8

**Published:** 2025-04-03

**Authors:** Xiuxing Liu, Chenyang Gu, Jianjie Lv, Qi Jiang, Wen Ding, Zhaohao Huang, Yidan Liu, Yuhan Su, Chun Zhang, Zhuping Xu, Xianggui Wang, Wenru Su

**Affiliations:** 1 State Key Laboratory of Ophthalmology, Zhongshan Ophthalmic Center, Sun Yat-Sen University, Guangdong Provincial Key Laboratory of Ophthalmology and Visual Science, Guangzhou, 510060 China; 2 Guangzhou Women and Children’s Medical Center, Guangzhou Medical University, Guangzhou, 510623 China; 3 Department of Clinical Medicine, Zhongshan School of Medicine, Sun Yat-Sen University, Guangzhou, 510060 China; 4 Department of Ophthalmology, West China Hospital, Sichuan University, ChengduSichuan, 610041 China; 5 Eye Center of Xiangya Hospital, Central South University, Changsha, 410078 China; 6 Hunan Key Laboratory of Ophthalmology, Xiangya Hospital, Central South University, Changsha, 410078 China

**Correction: Journal of Neuroinflammation (2023) 20:144** 10.1186/s12974-023-02829-3

In this article [1], there was an error in the pathology image for the naive group in Figure S1B. For completeness and transparency, the old incorrect Figure S1 and the correct Figure S1 are displayed below.

Incorrect Figure S1
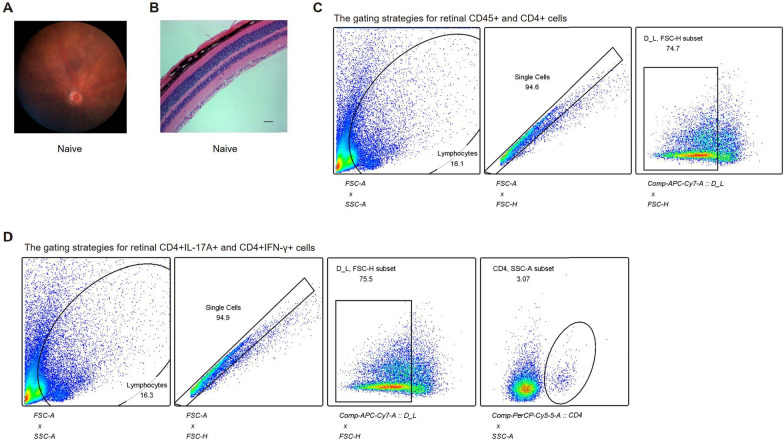


Correct Figure S1
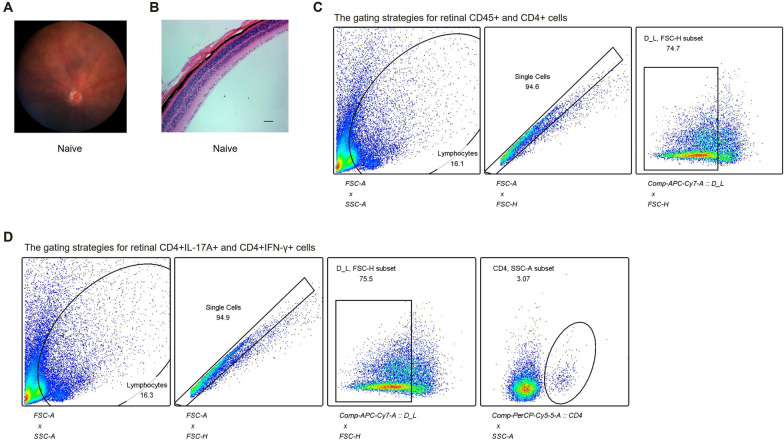


## References

[1] Liu X, Gu C, Lv J, Jiang Q, Ding W, Huang Z, Liu Y, Su Y, Zhang C, Xu Z, Wang X, Su W. Progesterone attenuates Th17-cell pathogenicity in autoimmune uveitis via Id2/Pim1 axis. J Neuroinflammation. 2023;20(1):144. 10.1186/s12974-023-02829-3. 37344856 10.1186/s12974-023-02829-3 PMC10286326

