# Supplementary material for: Progesterone attenuates Th17-cell pathogenicity in autoimmune uveitis via Id2/Pim1 axis
Source: J Neuroinflammation. 2023 Jun 21;20:144. doi: 10.1186/s12974-023-02829-3 (PMC10286326; doi:10.1186/s12974-023-02829-3)
Supplement: Supplementary file 1 — Additional file 1: Fig. S1. The effects of PRG on retinal cells during EAU. Fig. S2. The clustering strategies for scRNA-seq of CDLNs cells. Fig. S3. PRG reversed the EAU-induced inflammatory responses and PRG-related pathway disequilibrium. Fig. S4. The modulatory effects of PRG on CD4+ T cells. Fig. S5. The modulatory effects of PRG on Th17 cells. Fig. S6. The modulatory effects of PRG on Id2/Pim1 pathway in Th17 cells. [file 12974_2023_2829_MOESM1_ESM.pdf]

Additional files

Additional file 1: Table

Table S1. Cell clustering strategy in scRNA-seq.

Table S2. The differential expression analysis in Treg of Figure 4F-G.

Table S3. The differential expression analysis in Th17 (**A**) and Treg (**B**) of Figure 5A.

Table S4. The expression correlation analysis of downregulated rescue-DEGs of Th17 cells and the genes related to T cell activation, Th17 cell differentiation, IL-17 signaling pathway.

Additional file 2: Figure and Legend

Fig. S1. The effects of PRG on retinal cells during EAU.

A. The representative fundus image of naive group.

B. The representative H&E-stained image of naive group. Scale bars: 50 μm.

C. The flow cytometry histograms showing the gating strategies for retinal CD45+ and CD4+ cells.

D. The flow cytometry histograms showing the gating strategies for retinal CD4+ IL-17A+ Th17 and CD4+ IFN-γ+ Th1 cells.

Fig. S2. The clustering strategies for scRNA-seq of CDLNs cells.

A. The image showing the size of CDLNs among three groups.

B. The heatmap showing scaled expression of discriminative gene sets for each cell type.

C. t-SNE plot showing the immune cell types of each group in scRNA-seq.

Volcano plot showing the EAU-DEGs (**D**) or PRG-DEGs (**E**) in MONO.

Volcano plot showing the EAU-DEGs (**F**) or PRG-DEGs (**G**) in CD4.

Volcano plot showing the EAU-DEGs (**H**) or PRG-DEGs (**I**) in BC.

J. Volcano plot showing the up or downregulated PRG-DEGs in NEU.

K. Volcano plot showing the up or downregulated PRG-DEGs in TBC.

Fig. S3. PRG reversed the EAU-induced inflammatory responses and PRG-related pathway disequilibrium.

A. The heatmap showing the relative levels of genes related to PRG pathways among the

immune cell types.

B. The heatmap showing the relative levels of genes related to PRG pathways among three groups.

C. Bar chart showing the serum PRG levels of the three groups.

D. Violin plot showing the expression levels of *Fos*, *Cxcr4*, *Ly6a*, *Lef1*, *Jun*, *S100a13*, *Pim1* and *Tgfbr1* among three groups.

E. Volcano plot showing the rescue-DEGs in PDC cells.

F. Volcano plot showing the rescue-DEGs in CDC cells.

G. t-SNE plot (left) showing the BC subsets and the heatmap (right) showing scaled expression of discriminative gene sets for BC subsets.

Fig. S4. The modulatory effects of PRG on CD4+ T cells.

A. Representative GO biological process and pathways enriched in downregulated rescue-DEGs in CD4.

B. The heatmap showing the relative levels of discriminative genes in CD4+ TC subsets.

C. Rose diagram showing the number of up and downregulated EAU-DEGs (up) or PRG-DEGs (down) in CD4+ TC subsets.

D. The flow cytometry histograms showing the gating strategies for CDLNs CD4+ FOXP3+ Treg cells.

E. The flow cytometry histograms (left) and column charts (right) showing the percentage of CDLNs CD4+ FOXP3+ cells among three groups (n = 5/group).

F. The flow cytometry histograms showing the gating strategies for the expression of TGFBR2, BACH2, and IL-10 in CDLNs CD4+ FOXP3+ cells.

G. The flow cytometry histogram showing the Fluorescence Minus One (FMO) of BACH2.

H. The flow cytometry histograms (left) and column charts (right) showing the level of IL-10 in CD4+ FOXP3+ Treg cells among three groups (n = 5/group).

Significance in **E, H** was calculated using one-way ANOVA test; $^*P < 0.05$, $^{**}P < 0.01$, $^{***}P < 0.001$, $^{****}P < 0.0001$.

Fig. S5. The modulatory effects of PRG on Th17 cells.

A. Representative GO biological process and pathways enriched in downregulated rescue-DEGs in Th17.

B. UpSet plot showing the interactions of downregulated rescue-DEGs among CD4+ TC subsets.

C. Venn diagram showing the interaction of downregulated rescue-DEGs in Th17 and target genes of TFs.

D. Representative GO biological process and pathways involved by *Id2*.

The flow cytometry histograms showing the gating strategies for CDLNs CD4+ IL-17A+ cells (**E**), and the expression of Pim1, Id2, IL-23R, and GM-CSF in CDLNs CD4+ IL-17A+ cells (**F**).

G. The flow cytometry histogram showing the Fluorescence Minus One (FMO) of Pim1 (left) and Id2 (right).

Fig. S6. The modulatory effects of PRG on Id2/Pim1 pathway in Th17 cells.

A. The heatmap showing the expression correlation of downregulated rescue-DEGs of Th17 cells and these genes related to T cell activation, Th17 cell differentiation, IL-17 signaling pathway. Then the correlation module involving *Id2* was screened.

The flow cytometry histograms (left) and column charts (right) showing the level of Id2 in CD4+ T cells (**B**) and Th17 cells (**C**) between $IRBP_{1\text{-}20}$ or $IRBP_{1\text{-}20}$+HELI groups (n = 5/group).

D. The flow cytometry histograms (left) and column charts (right) showing the percentage of CD4+ IL-17A+ Th17 cells among control, $IRBP_{1\text{-}20}$, $IRBP_{1\text{-}20}$+PRG and $IRBP_{1\text{-}20}$+PRG+HELI groups (n = 5/group).

The flow cytometry histograms (left) and column charts (right) showing the level of IL-23R (**E**) and GM-CSF (**F**) in CD4+ IL-17A+ Th17 cells among $IRBP_{1\text{-}20}$, $IRBP_{1\text{-}20}$+IL-23 and $IRBP_{1\text{-}20}$+IL-23+PRG groups (n = 5/group).

The flow cytometry histograms (left) and column charts (right) showing the level of Id2 (**G**) and Pim1 (**H**) in CD4+ IL-17A+ Th17 cells between Vehicle-AT and PRG-AT groups (n = 5/group).

Significance in **B-C, G-H** was calculated using two-tailed unpaired Student's t-test; significance in **D-F** was calculated using one-way ANOVA test; $^*P < 0.05$, $^{**}P < 0.01$, $^{***}P < 0.001$, $^{****}P < 0.0001$.

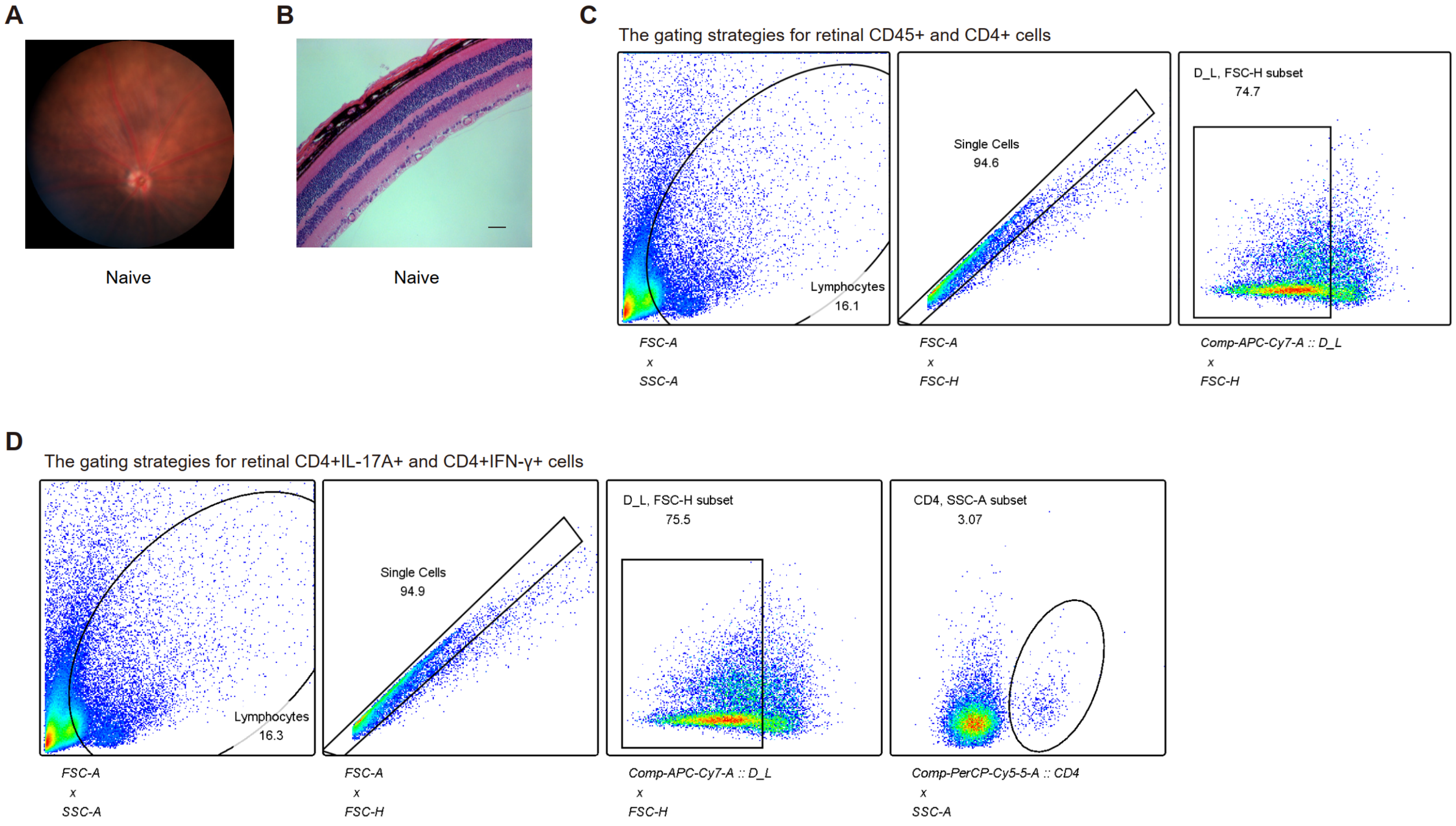
A
Naive
B
Naive
C
The gating strategies for retinal CD45+ and CD4+ cells
Lymphocytes
16.1
FSC-A
x
SSC-A
Single Cells
94.6
FSC-A
x
FSC-H
D_L, FSC-H subset
74.7
Comp-APC-Cy7-A :: D_L
x
FSC-H
D
The gating strategies for retinal CD4+IL-17A+ and CD4+IFN-γ+ cells
Lymphocytes
16.3
FSC-A
x
SSC-A
Single Cells
94.9
FSC-A
x
FSC-H
D_L, FSC-H subset
75.5
Comp-APC-Cy7-A :: D_L
x
FSC-H
CD4, SSC-A subset
3.07
Comp-PerCP-Cy5-5-A :: CD4
x
SSC-A

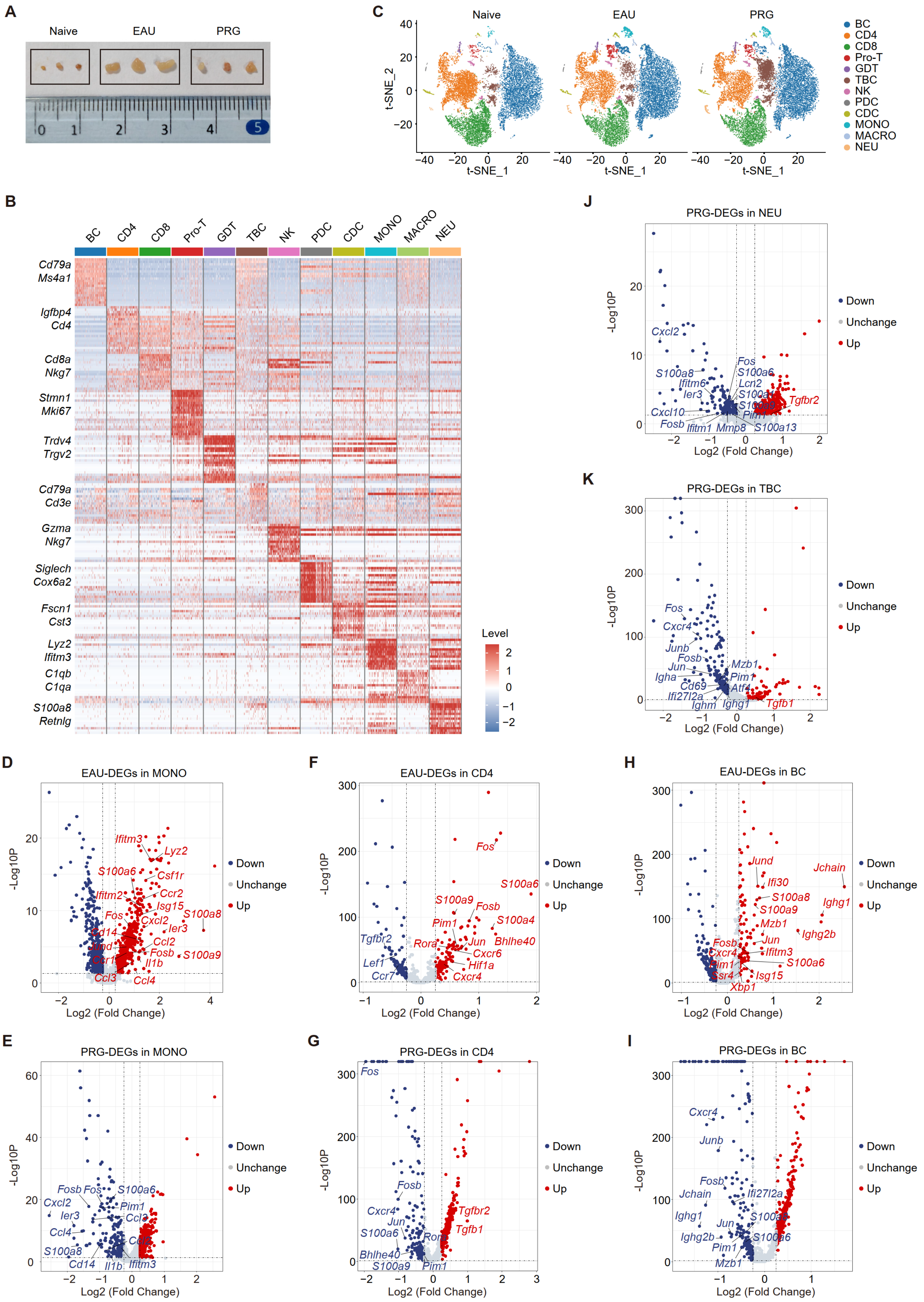
A
Naive
EAU
PRG
B
BC CD4 CD8 Pro-T GDT TBC NK PDC CDC MONO MACRO NEU
Cd79a Ms4a1
Igfbp4 Cd4
Cd8a Nkg7
Stmn1 Mki67
Trdv4 Trgv2
Cd79a Cd3e
Gzma Nkg7
Siglech Cox6a2
Fscn1 Cst3
Lyz2 Ifitm3
C1qb C1qa
S100a8 Retnlg
Level
C
t-SNE_1
t-SNE_2
BC CD4 CD8 Pro-T GDT TBC NK PDC CDC MONO MACRO NEU
D
EAU-DEGs in MONO
E
PRG-DEGs in MONO
F
EAU-DEGs in CD4
G
PRG-DEGs in CD4
H
EAU-DEGs in BC
I
PRG-DEGs in BC
J
PRG-DEGs in NEU
K
PRG-DEGs in TBC
-Log10P
Log2 (Fold Change)
Down
Unchange
Up

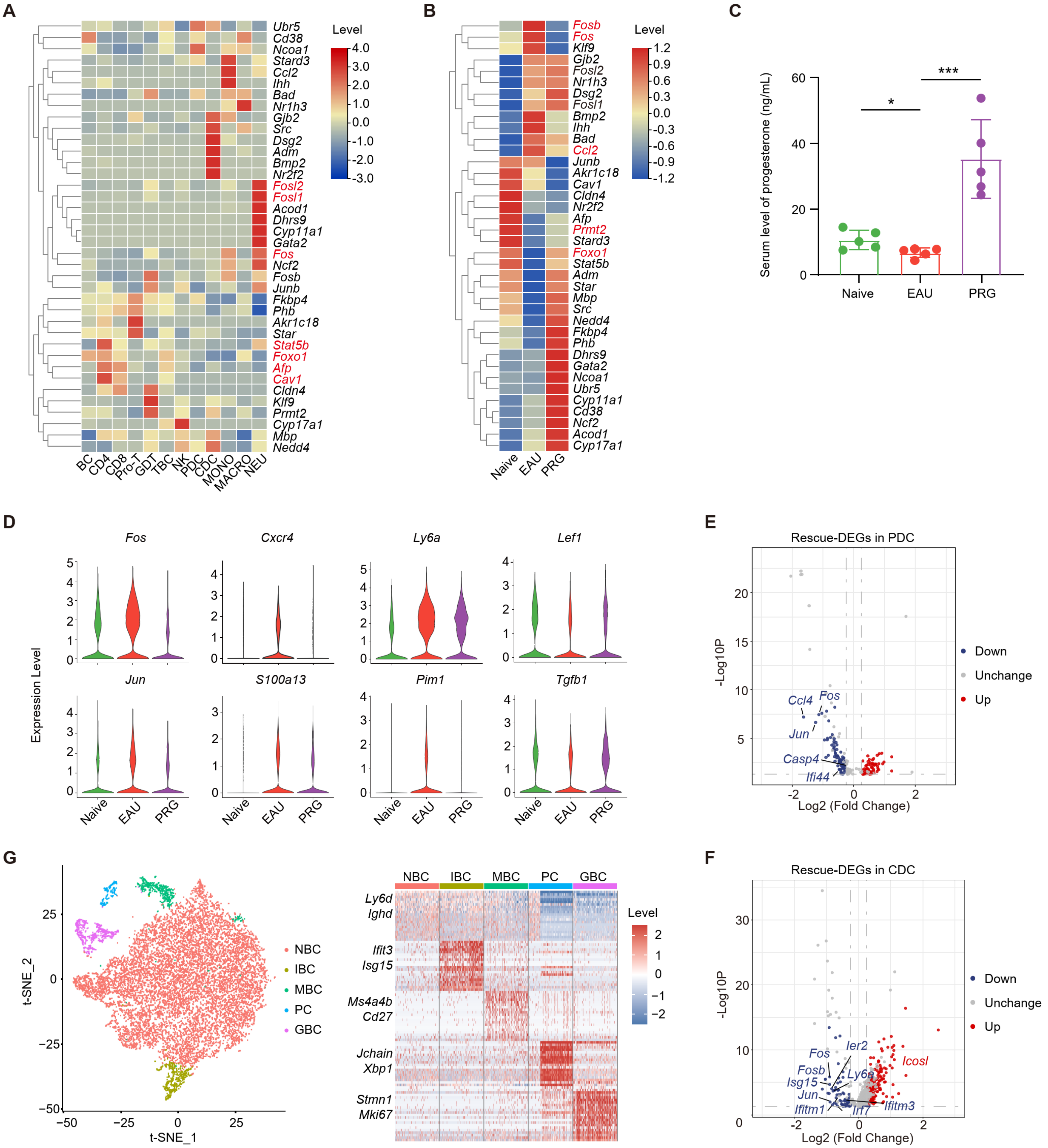
A
Level
4.0
3.0
2.0
1.0
0.0
-1.0
-2.0
-3.0
Ubr5
Cd38
Ncoa1
Stard3
Ccl2
Ihh
Bad
Nr1h3
Gjb2
Src
Dsg2
Adm
Bmp2
Nr2f2
Fosl2
Fosl1
Acod1
Dhrs9
Cyp11a1
Gata2
Fos
Ncf2
Fosb
Junb
Fkbp4
Phb
Akr1c18
Star
Stat5b
Foxo1
Afp
Cav1
Cldn4
Klf9
Prmt2
Cyp17a1
Mbp
Nedd4
BC
CD4
CD8
Pro-T
GDT
TBC
NK
PDC
CDC
MONO
MACRO
NEU
B
Level
1.2
0.9
0.6
0.3
0.0
-0.3
-0.6
-0.9
-1.2
Fosb
Fos
Klf9
Gjb2
Fosl2
Nr1h3
Dsg2
Fosl1
Bmp2
Ihh
Bad
Ccl2
Junb
Akr1c18
Cav1
Cldn4
Nr2f2
Afp
Prmt2
Stard3
Foxo1
Stat5b
Adm
Star
Mbp
Src
Nedd4
Fkbp4
Phb
Dhrs9
Gata2
Ncoa1
Ubr5
Cyp11a1
Cd38
Ncf2
Acod1
Cyp17a1
Naive
EAU
PRG
C
Serum level of progesterone (ng/mL)
60
40
20
0
*
***
Naive
EAU
PRG
D
Expression Level
Fos
Cxcr4
Ly6a
Lef1
Jun
S100a13
Pim1
Tgfb1
Naive
EAU
PRG
E
Rescue-DEGs in PDC
-Log10P
Log2 (Fold Change)
Ccl4
Fos
Jun
Casp4
Ifi44
Down
Unchange
Up
F
Rescue-DEGs in CDC
-Log10P
Log2 (Fold Change)
Fos
Ier2
Fosb
Isg15
Ly6a
Jun
Ifitm1
Irf7
Ifitm3
Icosl
Down
Unchange
Up
G
t-SNE_2
t-SNE_1
NBC
IBC
MBC
PC
GBC
Level
Ly6d
Ighd
Ifit3
Isg15
Ms4a4b
Cd27
Jchain
Xbp1
Stmn1
Mki67

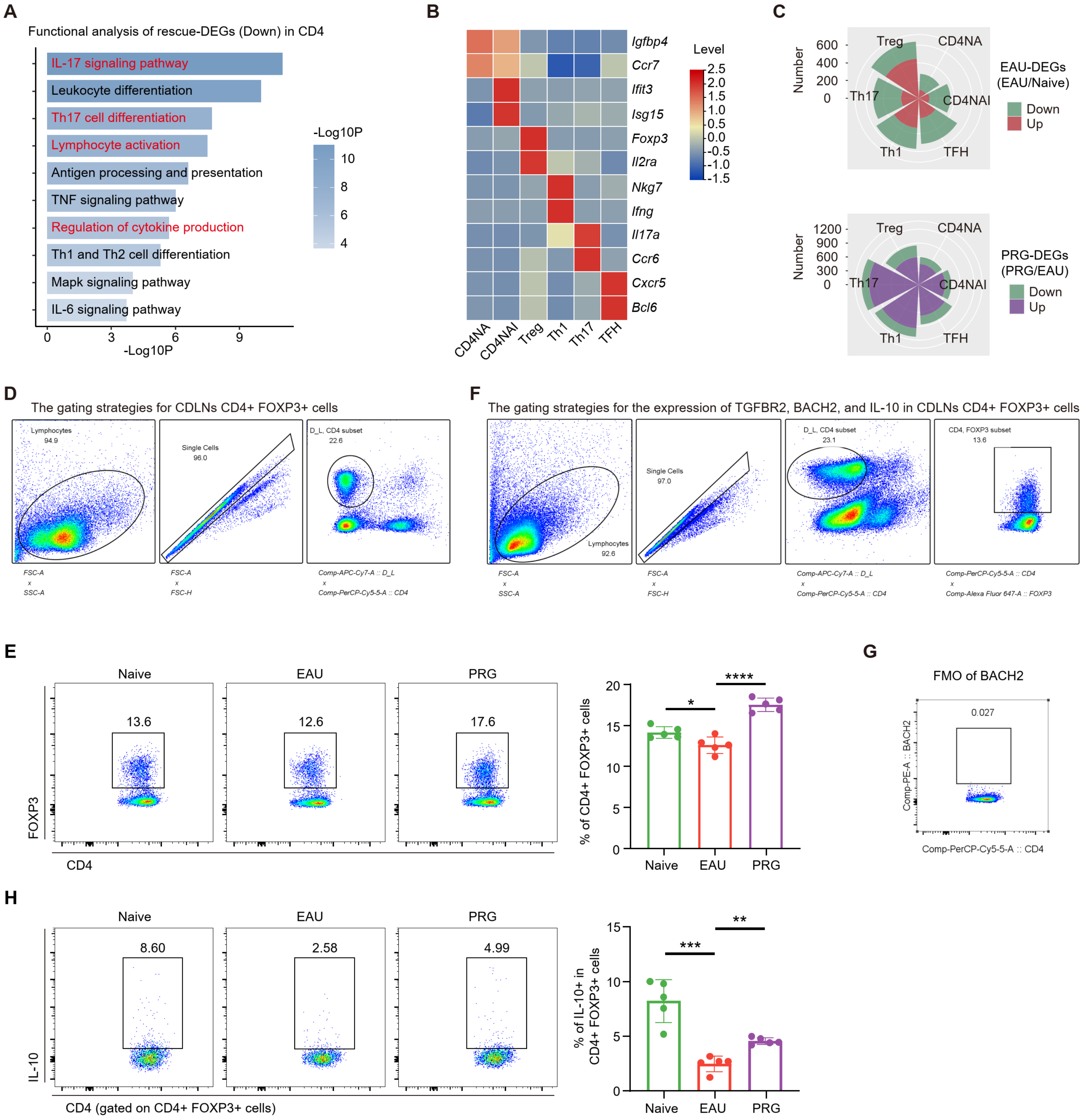

A
Functional analysis of rescue-DEGs (Down) in CD4
IL-17 signaling pathway
Leukocyte differentiation
Th17 cell differentiation
Lymphocyte activation
Antigen processing and presentation
TNF signaling pathway
Regulation of cytokine production
Th1 and Th2 cell differentiation
Mapk signaling pathway
IL-6 signaling pathway
-Log10P
B
Igfbp4
Ccr7
Ifit3
Isg15
Foxp3
Il2ra
Nkg7
Ifng
Il17a
Ccr6
Cxcr5
Bcl6
Level
CD4NA
CD4NAI
Treg
Th1
Th17
TFH
C
Number
EAU-DEGs (EAU/Naive)
PRG-DEGs (PRG/EAU)
Down
Up
D
The gating strategies for CDLNs CD4+ FOXP3+ cells
Lymphocytes 94.9
Single Cells 96.0
D_L, CD4 subset 22.6
F
The gating strategies for the expression of TGFBR2, BACH2, and IL-10 in CDLNs CD4+ FOXP3+ cells
Lymphocytes 92.6
Single Cells 97.0
D_L, CD4 subset 23.1
CD4, FOXP3 subset 13.6
E
Naive
EAU
PRG
13.6
12.6
17.6
FOXP3
CD4
% of CD4+ FOXP3+ cells
G
FMO of BACH2
0.027
Comp-PE-A :: BACH2
Comp-PerCP-Cy5-5-A :: CD4
H
8.60
2.58
4.99
IL-10
CD4 (gated on CD4+ FOXP3+ cells)
% of IL-10+ in CD4+ FOXP3+ cells

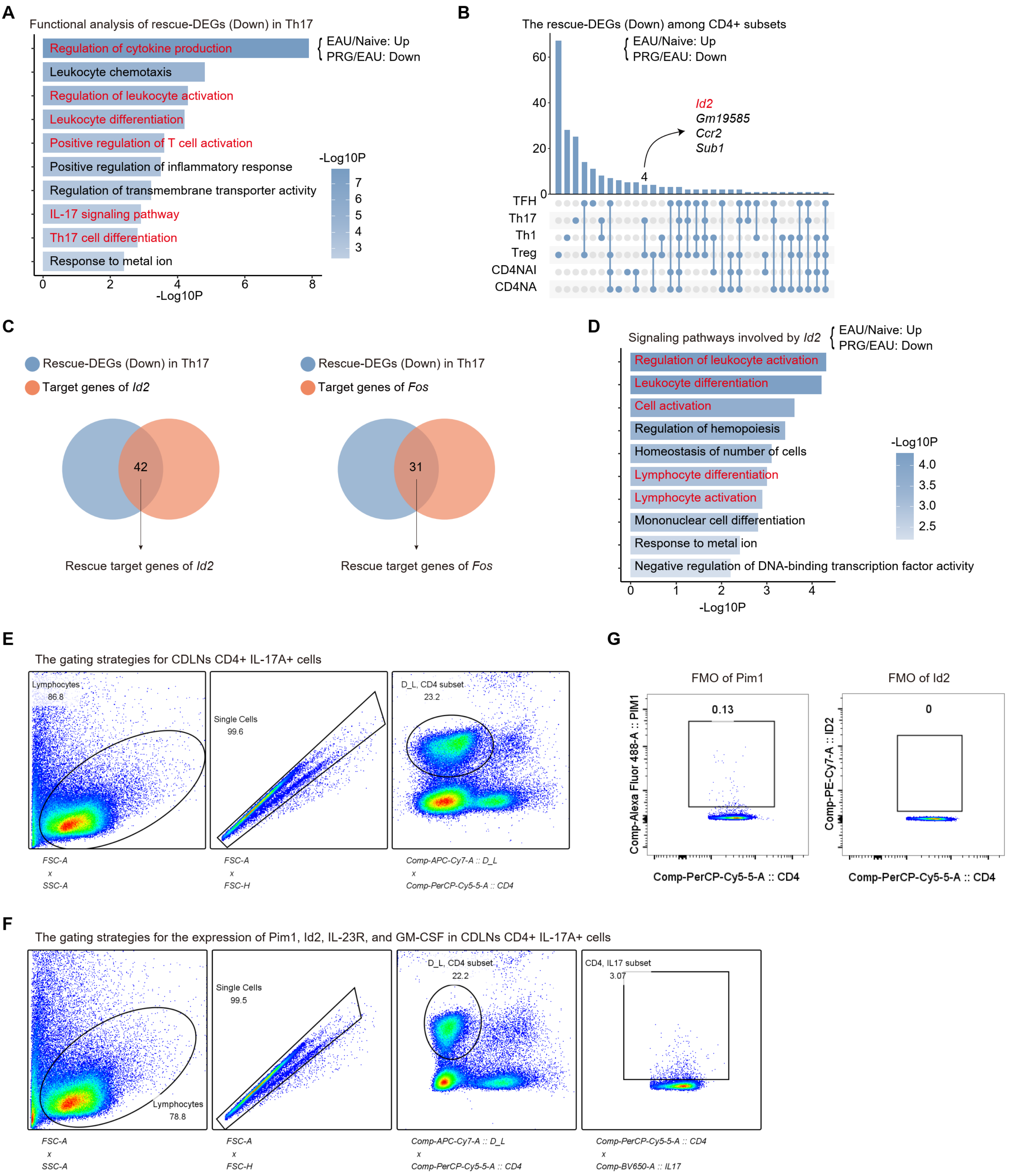
A
Functional analysis of rescue-DEGs (Down) in Th17
Regulation of cytokine production
Leukocyte chemotaxis
Regulation of leukocyte activation
Leukocyte differentiation
Positive regulation of T cell activation
Positive regulation of inflammatory response
Regulation of transmembrane transporter activity
IL-17 signaling pathway
Th17 cell differentiation
Response to metal ion
EAU/Naive: Up
PRG/EAU: Down
-Log10P
B
The rescue-DEGs (Down) among CD4+ subsets
EAU/Naive: Up
PRG/EAU: Down
Id2
Gm19585
Ccr2
Sub1
TFH
Th17
Th1
Treg
CD4NAI
CD4NA
C
Rescue-DEGs (Down) in Th17
Target genes of Id2
Target genes of Fos
42
31
Rescue target genes of Id2
Rescue target genes of Fos
D
Signaling pathways involved by Id2
EAU/Naive: Up
PRG/EAU: Down
Regulation of leukocyte activation
Leukocyte differentiation
Cell activation
Regulation of hemopoiesis
Homeostasis of number of cells
Lymphocyte differentiation
Lymphocyte activation
Mononuclear cell differentiation
Response to metal ion
Negative regulation of DNA-binding transcription factor activity
-Log10P
E
The gating strategies for CDLNs CD4+ IL-17A+ cells
Lymphocytes
86.8
Single Cells
99.6
D_L, CD4 subset
23.2
FSC-A x SSC-A
FSC-A x FSC-H
Comp-APC-Cy7-A :: D_L x Comp-PerCP-Cy5-5-A :: CD4
G
FMO of Pim1
FMO of Id2
0.13
0
Comp-Alexa Fluor 488-A :: PIM1
Comp-PE-Cy7-A :: ID2
Comp-PerCP-Cy5-5-A :: CD4
F
The gating strategies for the expression of Pim1, Id2, IL-23R, and GM-CSF in CDLNs CD4+ IL-17A+ cells
Single Cells
99.5
Lymphocytes
78.8
D_L, CD4 subset
22.2
CD4, IL17 subset
3.07
FSC-A x SSC-A
FSC-A x FSC-H
Comp-APC-Cy7-A :: D_L x Comp-PerCP-Cy5-5-A :: CD4
Comp-PerCP-Cy5-5-A :: CD4 x Comp-BV650-A :: IL17

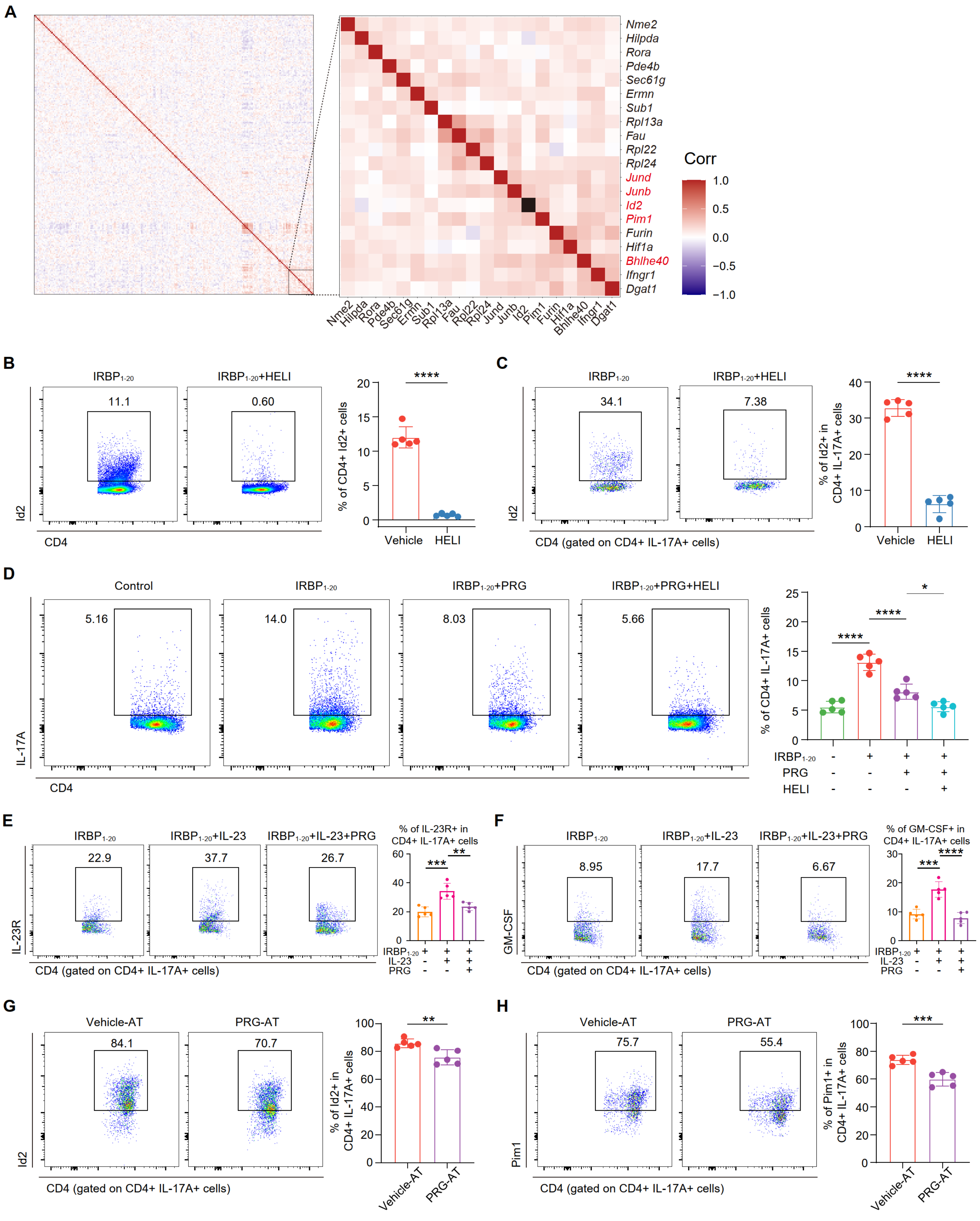
A
Nme2
Hilpda
Rora
Pde4b
Sec61g
Ermn
Sub1
Rpl13a
Fau
Rpl22
Rpl24
Jund
Junb
Id2
Pim1
Furin
Hif1a
Bhlhe40
Ifngr1
Dgat1
Corr
1.0
0.5
0.0
−0.5
−1.0
B
IRBP1-20
IRBP1-20+HELI
11.1
0.60
Id2
CD4
% of CD4+ Id2+ cells
Vehicle
HELI
****
C
34.1
7.38
CD4 (gated on CD4+ IL-17A+ cells)
% of Id2+ in CD4+ IL-17A+ cells
D
Control
IRBP1-20+PRG
IRBP1-20+PRG+HELI
5.16
14.0
8.03
5.66
IL-17A
% of CD4+ IL-17A+ cells
PRG
HELI
E
IRBP1-20+IL-23
IRBP1-20+IL-23+PRG
22.9
37.7
26.7
IL-23R
% of IL-23R+ in CD4+ IL-17A+ cells
IL-23
F
8.95
17.7
6.67
GM-CSF
% of GM-CSF+ in CD4+ IL-17A+ cells
G
Vehicle-AT
PRG-AT
84.1
70.7
% of Id2+ in CD4+ IL-17A+ cells
H
75.7
55.4
Pim1
% of Pim1+ in CD4+ IL-17A+ cells
